# Non-canonical DNA structures: Diversity and disease association

**DOI:** 10.3389/fgene.2022.959258

**Published:** 2022-09-05

**Authors:** Aparna Bansal, Shikha Kaushik, Shrikant Kukreti

**Affiliations:** ^1^ Nucleic Acid Research Lab, Department of Chemistry, University of Delhi, Delhi, India; ^2^ Department of Chemistry, Hansraj College, University of Delhi, Delhi, India; ^3^ Department of Chemistry, Rajdhani College, University of Delhi, New Delhi, India

**Keywords:** non-canonical DNA, G-quadruplex, triplex, cruciform, Z-DNA

## Abstract

A complete understanding of DNA double-helical structure discovered by James Watson and Francis Crick in 1953, unveil the importance and significance of DNA. For the last seven decades, this has been a leading light in the course of the development of modern biology and biomedical science. Apart from the predominant B-form, experimental shreds of evidence have revealed the existence of a sequence-dependent structural diversity, unusual non-canonical structures like hairpin, cruciform, Z-DNA, multistranded structures such as DNA triplex, G-quadruplex, i-motif forms, etc. The diversity in the DNA structure depends on various factors such as base sequence, ions, superhelical stress, and ligands. In response to these various factors, the polymorphism of DNA regulates various genes *via* different processes like replication, transcription, translation, and recombination. However, altered levels of gene expression are associated with many human genetic diseases including neurological disorders and cancer. These non-B-DNA structures are expected to play a key role in determining genetic stability, DNA damage and repair *etc*. The present review is a modest attempt to summarize the available literature, illustrating the occurrence of non-canonical structures at the molecular level in response to the environment and interaction with ligands and proteins. This would provide an insight to understand the biological functions of these unusual DNA structures and their recognition as potential therapeutic targets for diverse genetic diseases.

## Introduction

**FIGURE 1:**
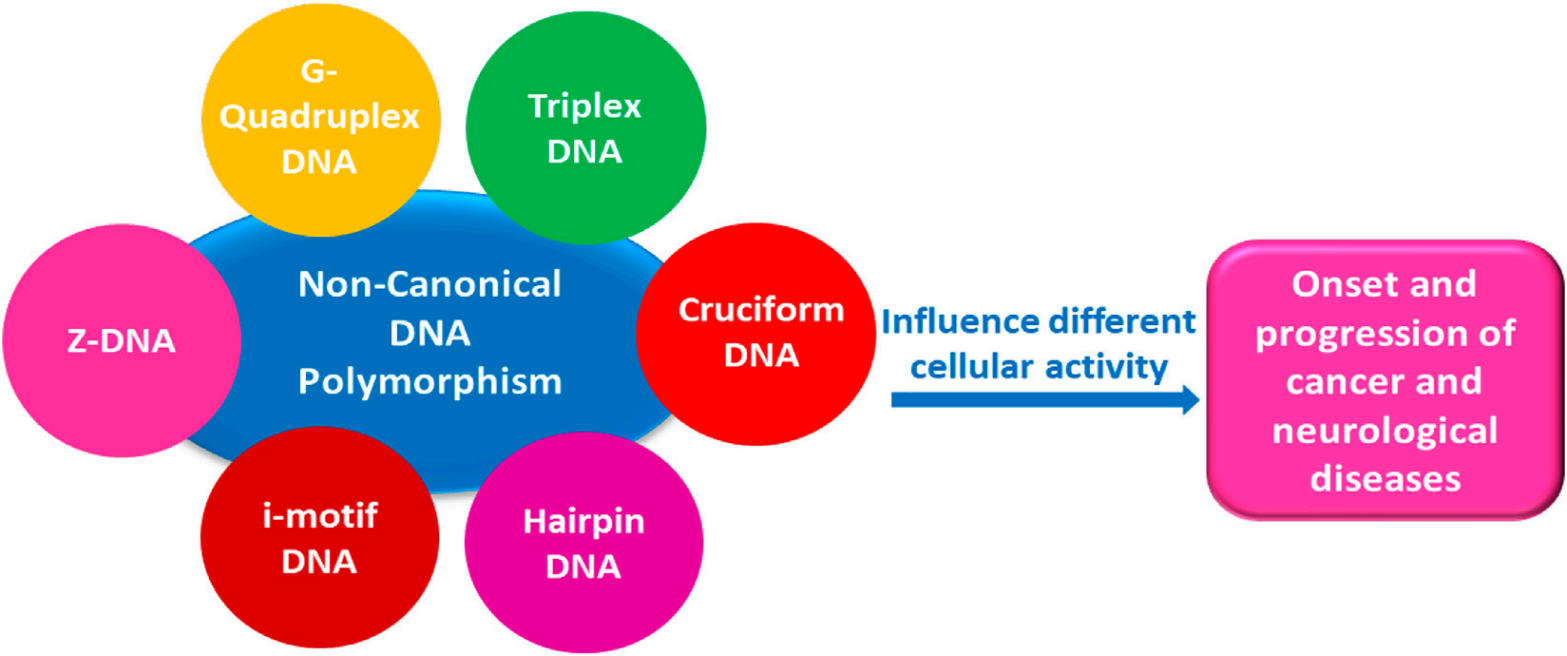
Schematic representation of DNA non-canonical structures and the diseases associated.

**FIGURE 2:**
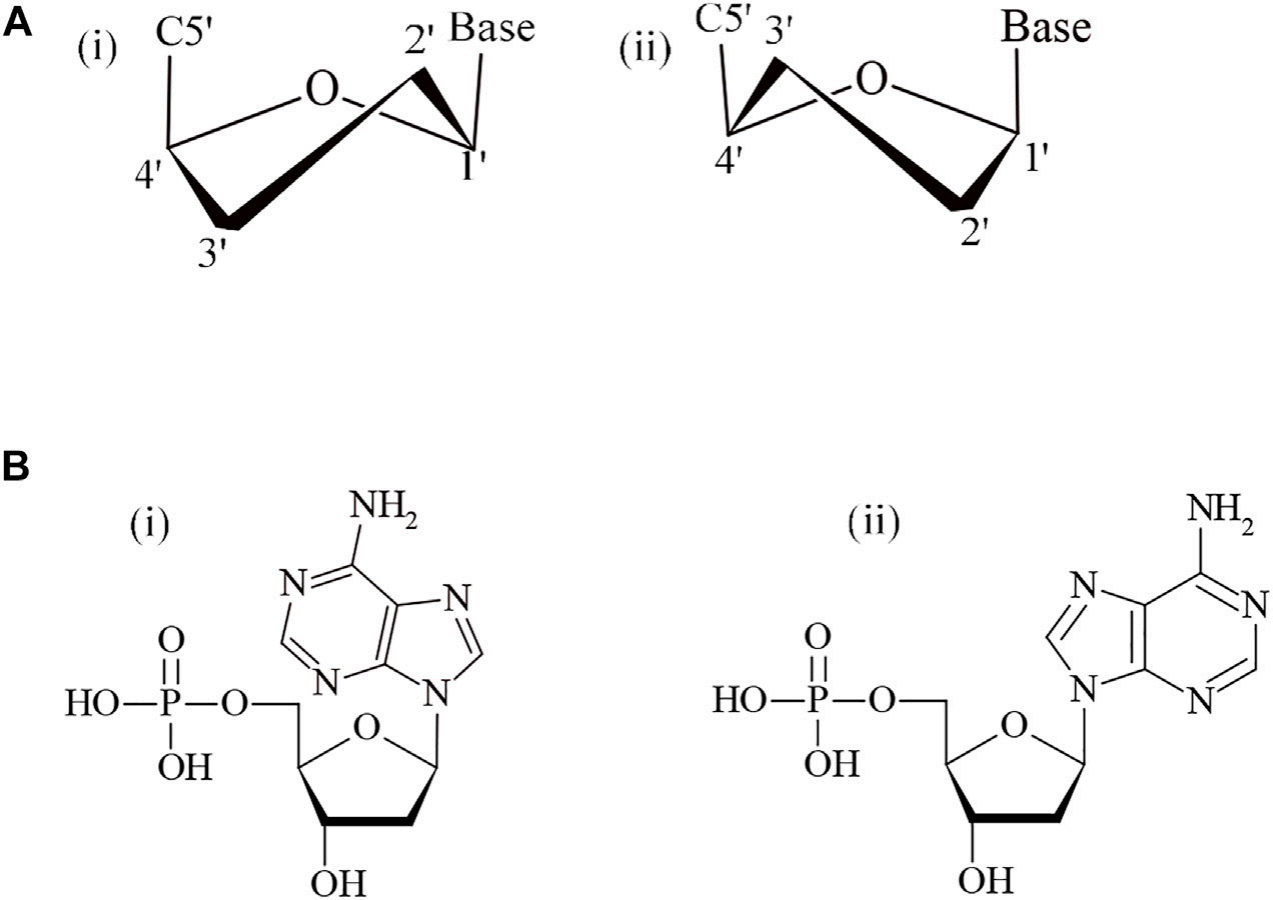
**(A)** Sugar pucker in DNA (i) C2′-endo and (ii) C3′-endo. **(B)** N-glycosidic bond conformations in DNA (i) syn and (ii) anti.

The voyage of studying DNA architecture is continued since after its discovery by James Watson and Francis Crick in 1953. The first B-DNA structure proposed by Watson–Crick is proven as a milestone in the history of science and paved the way for exploring and understanding of all life processes. Even after completing almost seven decades, research in this field astonishes fast, and each new day emerges with gripping information. Structurally, DNA is a flexible molecule. Watson–Crick’s rules of hybridization define secondary structures of nucleic acids. However, a variety of DNA and RNA structures do not rely on the simple A-T/U, GC base pairing, and disobey the Watson–Crick canon, and they are described as non-canonical. Nonetheless, the potential to adopt various non-canonical DNA structures depends on many factors like nucleotide sequence, hydration, ionic strength, and ligand, etc. A-DNA and B-DNA are the most commonly studied form of DNA but other non-canonical structures like Z-DNA, hairpin/cruciform, triplex, G-quadruplex, and i-motif are also well established as given in Figure 1 (Pandya et al., 2021). The topological diversity of DNA is also attributed to various sugar and backbone conformational variables, the directionality of the glycosidic bonds and steric extensions, along with different base-pairing elasticities (Kaushik et al., 2011). In the right-handed B-DNA, the sugar-base linkage (N-glycosidic bond) is normally anti and the sugar conformation is C2′-endo, whereas in the A-form DNA the sugar conformation is generally C3′-endo (Figure 2A). The Z-DNA, a structure favored by alternating GC-rich sequences, is different from A- and B-form DNAs as the purine (G) here adopts a syn orientation with C3′-endo pucker, whereas the pyrimidine residue (C) acquires anti-conformation and the sugar adopts the C2′-endo pucker (Figure 2B). The variation in sugar pucker and orientation of N-glycosidic bond affects the shape of duplex, widths, and depths of grooves, backbone hydration, and intrastrand phosphate–phosphate distances (Bothe et al., 2012; Egli 2019).

**FIGURE 3:**
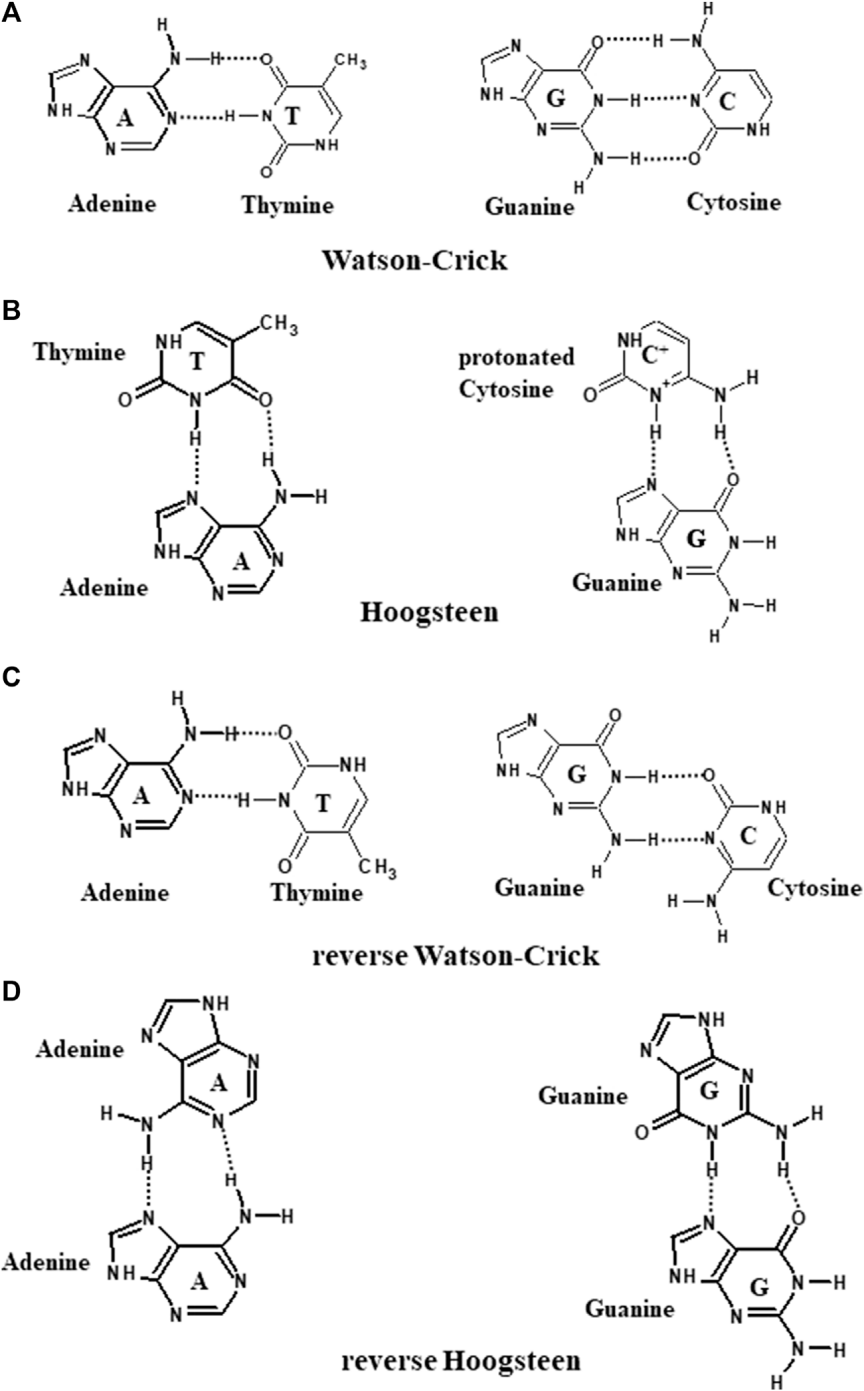
Schematic representation of **(A)** Watson–Crick, **(B)** Hoogsteen, **(C)** reverse Watson–Crick, and **(D)** reverse Hoogsteen hydrogen bonding patterns.

An assortment of possible hydrogen bonding patterns that contributes to the formation of a bouquet of DNA structures is shown in Figure 3 (Kaushik et al., 2016).

**FIGURE 4:**
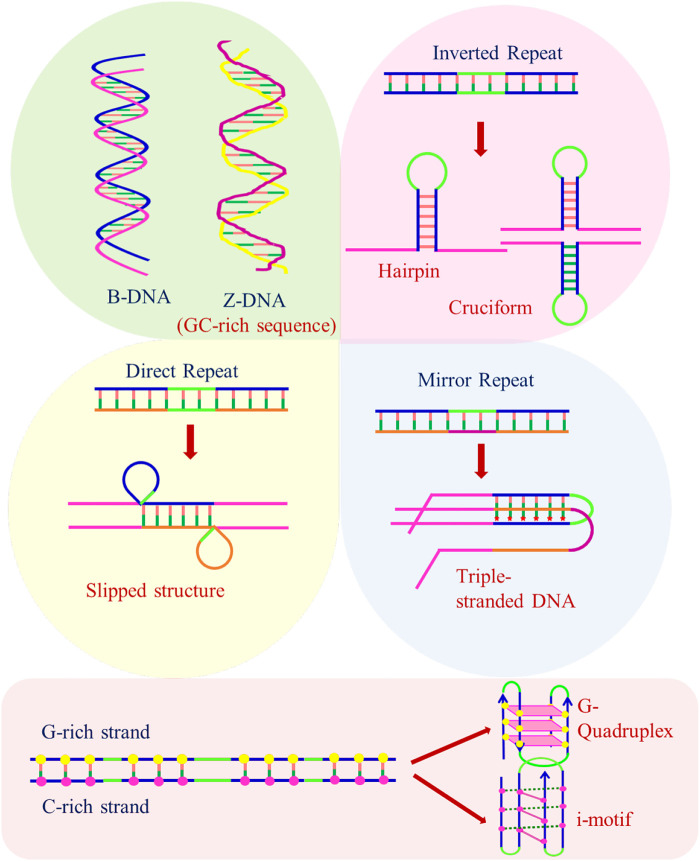
Structure of B-DNA and non-B-DNA.

These alternative/non-canonical/non-B-DNA structures have been found to form at particular repetitive sequences such as direct repeats, mirror repeats, inverted repeats, and short tandem repeats and play very important biological roles. Z-DNA requires an alternating purine–pyrimidine/GC-rich sequence, H-DNA requires a mirror repeat oligopurine sequence, cruciforms require inverted repeat sequences, G-quadruplexes require a contiguous stretch of guanines, while i-motif requires stretches of cytidines (Figure 4) (Georgakopoulos-Soares et al., 2018). A plethora of studies reveal that these non-B-DNA structures are associated with mutability and contribute to different types of cancer. Georgakopoulos-Soares et al. (2018) explored the structural motifs of these structures in the human genome and found that these non-B-DNA motifs are distributed non-uniformly and are extensively present at different chromosome regions. They can cause genetic instability in the presence/absence of DNA damage with/without replication and are also the hot spots of chromosomal breaks, homologous recombination, and gross chromosomal rearrangements (Wells 2007; Rajeshwari 2012; Wang and Vasquez 2014; Georgakopoulos-Soares et al., 2018).

The human genome consists of several potential sequences, capable of forming numerous non-canonical structures such as hairpin, Z-DNA, triplex, G-quadruplexes, and i-motifs. The formation of such structures in disease-related genes inhibit or dysregulate the essential biological processes and are considered as the endogenous sources of genomic instability (Wang and Vasquez 2004; Wang et al., 2008). They are also involved in many cellular processes such as DNA combination, epigenetic regulation of chromatin, and regulation (inhibition or promotion) of gene expression by transcription and translation (Tateishi-Karimata and Sugimoto 2020, 2021). These non-canonical structures play a role in cancer progression by perturbing the normal processes of central dogma. For example, DNA triplex and G-quadruplex structure formation may regulate the expression of cancer-related gene *via* these structures and thus inhibit transcriptional activity in the c-myc promoter region (Kim et al., 1998; Siddiqui-Jain et al., 2002). Several other studies have also shown that triplex-based approach can be employed to block transcription of various disease-causing genes. One interesting study on similar lines is DNA triplex-mediated inhibition of Ets2 transcription that results in growth inhibition and apoptosis in human prostate cancer cells (Carbone et al., 2004). Akhtar and Rajeshwari (2017) have reported that triplex formation selectively inhibits high-mobility group A1 proteins (HMGA1) expression and induce apoptosis in human cervical cancer. These studies have suggested that triplex-forming oligonucleotides (TFOs) that selectively target triplex-forming sequences in the promoter site of various oncogenes make attractive drug targets against anticancer therapeutics and would help the scientific community to synthesize/discover modulable ligands that show higher affinity for such triple-stranded structures and act as promising anticancer agents.

Recent studies have made known that the non-canonical structures formed by different repeat motifs of neurodegenerative disease-causing genes contribute in dysfunctioning of transcription and translation and thus causes toxic proteins accumulation. Also, intracellular phase separation promoting transcription and protein assembly are managed by these non-canonical structures that further direct the progression of neurodegenerative disease and cancer (Tateishi-Karimata and Sugimoto 2021). Interestingly, significant progress has been made to determine the therapeutic role of non-canonical structures made from chemically modified nucleobases. Reports divulge that chemically modified nucleobases like methyl, halogen, and aryl modifications at the C8 position of purines, and the C5 of pyrimidines can form DNA non-canonical structures. These modified sequences are associated with disease-inducing genes and play an important role in biological functions (Balasubramaniyam et al., 2021).

Herein, we have tried to summarize the facts illustrated in the literature about different non-canonical structures formed by DNA and their association with various human diseases at the level of genome stability, transcriptional and translational regulation *etc*. Throughout this review, we modestly abridge the current state of knowledge about the consequences of compromised genetic stability due to the presence of various DNA repeats at unlike locations in the genome.

## Hairpin/cruciform secondary structures

Palindromic DNA sequences consist of inverted repeats (IRs), which are either adjacent (perfect palindrome) or separated by a spacer region (quasi-palindrome). These are sequences with internal symmetry such that they can switch between inter-strand and intra-strand base pairing. Palindromic sequences play a vital role in regulating various biological processes and are widely distributed throughout the genome. It has been found that if the palindrome is of sufficient length, these repeats consequently form hairpins if only single-stranded DNA is involved and a cruciform structure with two hairpins forms when a double-stranded DNA is involved. Each hairpin is represented by a stem with complementary paired inverted repeats and a loop (Mitas 1997; SvetecMiklenić and Svetec 2021).

Several studies have illustrated that the hairpin structure is formed during DNA replication by the single-stranded lagging strand, while the cruciform structure is generated by the gradual extrusion of double-stranded DNA at the center of palindrome. Cruciform structures that are first proposed by Platt et al. (1955) are the best-explored species of unusual DNA. Its formation involves intra-strand hydrogen bond formation between complementary bases and disruption of intra-strand hydrogen bond of inverted repeats. It has been observed that there is a pre-requisite of at least ten base pair at the center of symmetry of an inverted repeat palindromic sequence to undergo unwinding before nucleation. This process of intra-strand fusion and inter-stand fission is driven by the energy provided by negative DNA supercoiling. It has been found that relaxation of one negative supercoil occurs for every 10.5 base pairs of the inverted repeats in cruciform formation. The unwinding step in the cruciform formation depends on many factors like temperature, base composition, ionic strength, and superhelical density (Sinden 1994). Numerous studies have revealed the interaction of cruciform with various proteins, which recognize various structural features like DNA crossover, four-way junctions, and curved DNA. These structural transitions take place during DNA replication and transcription and lead to the formation of alternative DNA structures. It has been reported that there are some proteins that specifically binds to cruciform structure and contribute in various processes like DNA repair, transcription, and replication. Brazda et al. has reviewed the cruciform protein interaction remarkably. They have explained well the cruciform-protein binding and their functions and thus categorized them into 1) junction resolving enzymes, 2) transcription factors and DNA repair proteins, 3) replication machinery, and 4) chromatin-associated proteins. They also explain the onset and progression of diseases caused by dysregulation of these proteins (Brázda et al., 2011).

The various genomes explored by Miklenic et al. including the human revealed its complex architecture that comprises different sequences, which play important roles in varied biological processes. Palindromic sequences are a potent source of genetic instability as they cause chromosome breakage (double-strand break, DSB), which further lead to genetic recombination, resulting in translocation, deletion, or gene amplification (Bissler 1998; Saada et al., 2021; SvetecMiklenić and Svetec 2021). Interestingly, the formation of hairpins and cruciform due to inter- and intra-strand base pairing of inverted repeats is associated with mutation and thus lead to several human genetic diseases, some of which are discussed in Table 1.

**TABLE 1:** Diseases associated with hairpin and cruciform structures.

S.No.	Disease	Characteristics	References
1	Hereditary angioneurotic edema	Autosomal dominant disease caused by a mutation involving imperfect inverted repeats reduces the production of functional C1 inhibitor (regulatory protein in inflammation), which leads to tissue edema of the skin and mucosal surfaces	Bissler et al. (1997)
2	Triplet repeat mediated disease (CTG•CAG triplet repeat)	Myotonic dystrophy type-1 (DM1), an autosomal dominant neuromuscular disease, is caused by the expansion of CTG•CAG triplet repeat (>50 repeats) in the 3′ UTR region of the DM protein kinase gene (DMPK) on chromosome 19	Bacolla et al. (2006)
			Izzo. et al. (2022)
3	Duchenne muscular dystrophy	X-linked recessive disease is mapped at dystrophin gene Xp21. ∼one out of three thousand male new born suffer from muscular dystrophy	Robinson et al. (1997)
4	Osteogenesis imperfecta	Autosomal recessive disease characterized by brittle bones. The disease is associated with mutation (deletion, insertion, and point mutation) in the type-1 procollagen gene	Bateman et al., 1989, Lamandé 1995, Chessler et al., 1993
5	Antithrombin deficiency	Mutation in the antithrombin gene leads to the development of venous thromboembolism at a young age	Davis III et al. (1993)
6	Silent serum cholinesterase	Autosomal recessive phenotype manifests an absence of enzyme activity due to a deficiency of human serum cholinesterase	Nogueira et al. (1990)
7	Lesch-Nyhan syndrome	Neurological disease that involves mutation, which inactivates the human hypoxanthine phosphoribosyl transferase (HPRT) gene. This syndrome intellectual disability, self-mutilation, polyathetosis, and an enhanced uric acid in serum	Gibbs et al. (1990)
8	Kearn-Sayre syndrome	This syndrome features retinal degeneration and a cardiac conduction block caused by a deletion in mitochondrion DNA	Remes et al., 1993
9	Biotinase deficiency	Autosomal recessive disorder with disability of recycling biotin. Thus, deficiency of biotin in the unavailability of functional biotinase shows clinical disorders like ataxia, seizures, and developmental delay coma	Pomponio et al. (1996)
10	Familial hypercholesterolemia	Autosomal dominant disease found in 1 out of 500 people caused by different types of mutation like deletion/insertion in the exon 8 of LDL receptor	Yamakawa-Kobayashi et al. (1994)

Moreover, genetic analysis has revealed that ∼80% of all inverted repeats in the human genome are short (< 100 bp) and enrich at translocation breakpoint in cancer and stimulate the double-strand break. While, the abundance of long inverted repeats (>500 bp) is rare in the human genome, they also contribute to deletion, recombination, and gene amplification. Different studies on the mutagenic potential of short IRs have demonstrated the formation of cruciform at short IRs, which stimulate DSB by stalling RNA replication forks that cleave the structure causing deletion. The study also explained well about the relative pathway between short IRs and human cancer points and establishes the hypothesis that these non-canonicals are involved in genetic instability, etiology, disease, and evolution (Lu et al., 2015; SvetecMiklenić and Svetec 2021).

As discussed, palindromic sequences are prone to cause chromosomal instability and lead to various diseases. Analysis revealed that breakpoints of palindrome-mediated translocations present at the center of palindromic AT-rich repeats (PATRRs) in the human genome are responsible for frequent non-Robertsonian translocation, which results in Emanuel syndrome and non-recurrent translocation (Kato et al., 2012; Saada et al., 2021). Hence, numerous reports give an account for various diseases associated with palindrome-mediated large deletions, interchromosomal insertions, and translocation, for example, εγ∆β thalassemia (Rooks et al., 2012), X-linked congenital hypertrichosis syndrome (Zhu et al., 2011), hereditary renal cell carcinoma (Kato et al., 2014) *etc*.

### Z-DNA polymorphic form

Z-DNA is the double-stranded left-handed DNA conformation. The existence of this polymorphic form of DNA was unexpected and so its discovery was quite accidental in 1970. Using the X-ray diffraction pattern and CD spectra, Mitsui et al. (1970) suggested the occurrence of left-handed conformation in poly d (I–C)•poly d (I–C) . Then, using the X-ray crystal structure of d (CpGpCpGpCpG), Alexander Rich and coworkers proposed the existence of the only known left-handed helical conformation of DNA, that is, Z-DNA in 1979 (Wang et al., 1979). It can be formed with alternating purine-pyrimidine dinucleotide repeat sequences like (GC)n. Interestingly, it has not found easy in case of (AT)n. Under special conditions of high salt concentration or presence of at least 10 A•T base pairs embedded between GC or GT can form Z-DNA (Klysik et al., 1988; Nejedly et al., 1989). DNA supercoiling has also been found crucial in the formation and stabilization of Z-DNA. The presence of negative supercoiling minimizes the salt concentration required to drive Z-conformation. Furthermore, the level of supercoiling also affects the length of the alternating purine–pyrimidine tract required for Z-DNA formation (Sinden 1994). It has been reported that the BZ junction (B- and Z-DNA meeting point) forms at both sides of Z-DNA-forming sites is important for alleviating torsional stress and stabilizing the Z-DNA (Subramani et al., 2019).

Z-DNA has a zig-zag sugar-phosphate backbone with alternating syn–anti conformation. The bases are not toward the center of the helix-like B-DNA, they are positioned toward the outside of the helix. The small distance between the negatively charged phosphates leads to the repulsion of the phosphates and causes destabilization of the Z-DNA. Hence, the presence of cations like Na^+^, K^+^, Rb^+^, Cs^+^, Li^+^, Mg^2+^, and Mn^2+^ stabilize the structure by shielding the negative charge. Some polyamines like spermine, spermidine, and putrescine also stabilize Z-DNA. (Sinden 1994; Gueron et al., 2000; Drozdzal et al., 2021).

Interestingly, research has evidenced the formation of Z-DNA and induces instability at the site of trinucleotide repeats (CAG, CGG, and GAC), which are associated with various neurodegenerative disorders like fragile X chromosome and skeletal dysplasia (Vorlickova et al., 2001; Renciuk et al., 2010; Khan et al., 2015). Also, Suram et al. (2002) showed the formation of Z-DNA signatures through CD spectra of severely affected Alzheimer’s disease DNA while the B-DNA conformation in normal, young, and aged brain DNA .

The dynamic nature of Z-DNA has always intrigued scientists to unveil different facts about Z-DNA. The flipping of B-DNA into Z-DNA without strand cleavage by negative superhelical stress has made this left-handed conformation the dominion of biology (Schwartz et al., 1999). It is believed that Z-DNA regulates the level of supercoiling, and thus plays a very important role in transcription, gene expression eliciting immunogenic responses *etc*. Z-DNA-forming sequences play a vital role in controlling the cellular processes like recombination, translocation, and deletion (Wang and Vasquez 2007). Spontaneous chromosomal breakage at the genomic hot spot can cause translocation-related human disease. Wang et al. demonstrated that Z-DNA-forming sequences in human tumors are the hot spots of chromosomal breakpoints. These Z-DNA-forming sequences were found to be responsible for genetic instability as they caused gene translocation in leukemias and lymphomas (Wang and Vasquez 2007). Furthermore, several other studies reported in the literature depicted the role of Z-DNA-forming sequences in chromosomal breakage and translocation in genes such as human BCL-2 (Adachi and Tsujimoto 1990; Seite et al., 1993), c-myc (Rimokh et al., 1991; Wölfl et al., 1995), and the SCL (Aplan et al., 1992), cancer-causing genes. Several immunoglobin-related genes ETV6 are associated with blood-related cancers. Also, Crohn’s disease, lupus erythematosus (SLE), amyotrophic lateral sclerosis (ALS), and polyradiculoneuritis are associated with the immunogenic behavior of Z-DNA (Ravichandran et al., 2019).

Z-DNA-forming DNA sequences generate large-scale deletions in mammalian cells (Wang et al., 2006). A correlation have been demonstrated between Z-DNA-forming DNA-segments and chromosome breakage hot spots, on tumor-related genes such as human c-myc, BCL-2, and the SCL gene, suggesting that Z-DNA may be implicated in chromosomal breakage and translocation. A study from Tsujimoto’s group has observed that numerous alternating purine–pyrimidine fragments surround the breakpoints in the 5′ flanking region of the BCL-2 gene. As per the Z-DNA-specific antibodies, the alternating purine–pyrimidine elements were constricted to the 5′ hot spot region. The information indicates to a potential role for Z-DNA production in chromosome translocations in the 5′ flanking region of the BCL-2 gene (Adachi and Tsujimoto 1990). Similarly, three sites in the human c-myc gene, Z-1 and Z-2 in the upstream promoter region and Z-3 at the juncture of the first intron and the second exon, have been found to generate Z-DNA in metabolically active nuclei. Z-DNA formation is only identified in all three elements by antibody binding when the gene is actively being transcribed. Negative supercoiling upstream of the gene is sufficient to stabilize the Z-DNA in Z-1, Z-2, and Z-3 site of the c-myc gene (Wölfl et al., 1995). Furthermore, Aplan et al. reported a T cell ALL (acute lymphoblastic leukemia) instance that additionally has a t (1; 3) (p34; p21) translocation, which damages the SCL locus and causes dysregulated SCL gene expression. A segment on chromosome 3 comprising alternating purine and pyrimidine repeats, forming a Z-DNA structure, is otherwise believed to be susceptible to recombination processes and is likewise disrupted by the t (1; 3) (Aplan et al., 1992).

Likewise, ADAM-12, a multidomain protein performs various functions like cell signaling, regulation of growth factors (heparin-binding EGF (HB-EGF), insulin-like growth factor binding protein-3 (IGFBP-3), and proteolysis. Ray et al. have demonstrated that there exists a highly conserved negative regulatory element (NRE) at the 5′-UTR of human ADAM-12 gene containing a stretch of dinucleotide repeat sequence, capable of forming Z-DNA. This NRE element was found to interact with Z-DNA-binding protein (hZαADAR1) and act as transcription repressor. The basic expressions of ADAM-12 are low in adults but it increases due to physiological conditions like during pregnancy in placenta. Interestingly, it is demonstrated that under normal physiological conditions, NRE transcriptional repressor capable of forming Z-DNA is required for ADAM-12 gene expression (Mochizuki et al., 2007; Ray et al., 2011). Several other studies have shown that overexpression of ADAM-12 causes pathological conditions detected in many tumor tissues like breast, liver, brain, and bone (Tian et al., 2002; Lendeckel et al., 2005; Kveiborg et al., 2008)

Among all the Z-DNA-binding proteins derived from different sources, double-stranded RNA adenosine deaminase 1 (ADAR1), vaccinia virus protein (E3L), and DLM-1 (also known as Z-DNA-binding protein-1, ZBP-1) are the most studied Z-DNA-binding proteins (Wang and Vasquez 2007). It has been found that all the three proteins bind to Z-DNA and Z-RNA through the same Zα domain. ADAR is an enzyme that deaminated the adenosine in ds RNA to produce inosine. This edit in the codon changes the protein sequence as the inosine is translated to guanosine and thus changes the expression of the gene they regulate (Soloman et al., 2017). E3L, an IFN resistance gene encoded by the vaccinia virus whose N-terminal domain has the sequence similarity to the Zα domain of ADAR-1 and DLAM-1. It has been depicted that E3L protein is required to inhibit an IFN-primed virus-induced necroptosis (Koehler et al., 2017). DLM-1 is a tumor-associated gene highly expressed in lymphatic tissues (Fu et al., 1999). Human Mendelian genetic studies by Alan Herbert reported the occurrence of rare genetic diseases- dyschromatosis symmetrica hereditaria (DSH), Aicardi–Goutieres syndrome (AGS), and bilateral striatal necrosis (BSN) produced by a mutant allele of the Zα domain (Herbert 2020).

## Non-canonical multistranded structures

### DNA triplex

**FIGURE 5:**
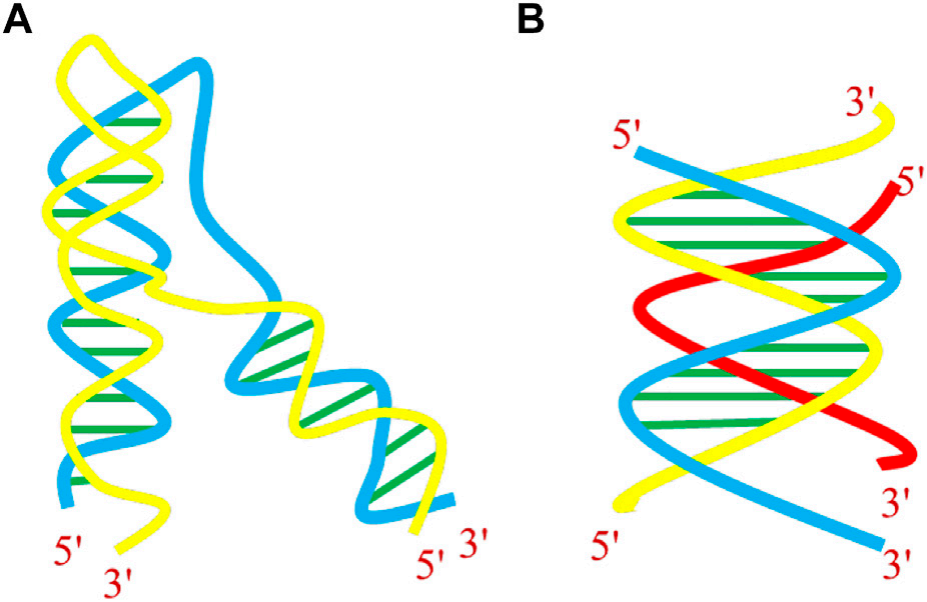
Helical representation of **(A)** intramolecular and **(B)** intermolecular DNA triplex.

Shortly after the discovery of DNA’s double-helical structure, the capability of nucleic acids to form triple-helical structures (triplexes) was established (Felsenfeld et al., 1957). DNA triplex, another non-B-DNA structure is formed when a single-stranded triplex-forming oligonucleotide (TFO) binds to the major groove of polypurine•polypyrimidine (Pu•Py) segments of a target duplex in a sequence-specific manner. This spurring work was the starting point for several studies, showing that purine strand of Watson–Crick duplex could bind to the third strand containing either purines or pyrimidines. The specificity and stability of the triple-helical structures are met by Hoogsteen or reverse Hoogsteen hydrogen bonding patterns, different from the classical Watson–Crick hydrogen bonding of B-DNA. As a prerequisite, the formation of triple-helical structures requires a polypurine•polypyrimidine tract, and many databases have been developed to predict the genome-wide locations of these triplex-forming sequences. Studies have shown that the mammalian genome contains numerous triplex-targeting sites (TTS), especially in 3′- untranslated region, 5′- of the promoter region, the gene linked with cell signaling and communication in the promoters, and within the transcribed regions of many genes (Bacolla et al., 2006; Buske et al., 2012; Jenjaroenpun et al., 2015). Literature is rich in reports that homopyrimidine and some homopurine tracts can form stable three-stranded complexes with corresponding Pu•Py sites by binding in the major groove of the target duplex (Sun and Helene 1993; Thuong and Helene 1993; Buchini and Leumann 2003). The third-strand binding to the DNA duplex can come from the same molecule, resulting in the formation of an intramolecular triplex or from a different molecule, termed an intermolecular triplex (Figure 5).

**FIGURE 6:**
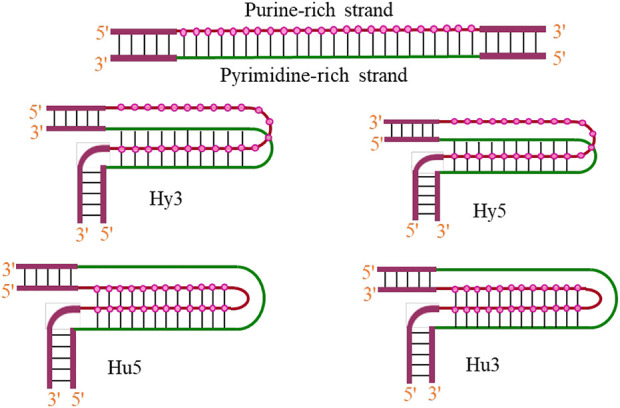
Possibilities of intramolecular triplex formation (H/*H-DNA).

Triplex formation arises from the fact that the base pairs of the duplex already involved in Watson–Crick hydrogen bonding, still have some hydrogen bond acceptor and donor sites, available for recognition of specific bases. Thanks to the hydrogen bonding ability of some nitrogenous bases. The third strand interacts through another surface of the Watson–Crick duplex, resulting in the formation of base triplets *via* the formation of Hoogsteen or reverse Hoogsteen hydrogen bonds. Intramolecular structures are formed when unwinding of homopurine•homopyrimidine mirror repeats within the DNA duplex allows one strand to fold back and be accommodated with the target duplex (Mirkin et al., 1987). Although, homopurine•homopyrimidine mirror repeats represent an ideal target for the formation of H-DNA *in vitro* (Wells et al., 1988; Frank-Kamenetskii and Mirkin 1995) nevertheless, these structures have also been reported to form within sequences those are neither homopurine•homopyrimidine nor mirror repeats (Dayn et al., 1992). Studies on DNA triplexes have shown that non-mirror repeat sequences can also form intramolecular triplexes (Klysik 1995), and such structures are often observed even with some mismatches (Belotserkovskii et al., 1990; Macaya et al., 1991). Intramolecular triplexes that exist *in vivo* are known as H-DNA, and depending on whether the third strand is pyrimidine- or purine-rich, these structures are known as H-DNA or *H-DNA, respectively. Such structures are preferentially formed under negative superhelical stress, acidic pH, and in presence of divalent cations (Kohwi and Kohwi-Shigematsu 1988). Four different intramolecular isomers of triplex DNA (Hy3, Hy5, Hu3, and Hu5) have been reported in the literature as shown in Figure 6 and are named on the basis of which half-element serves as the third strand.

**FIGURE 7:**
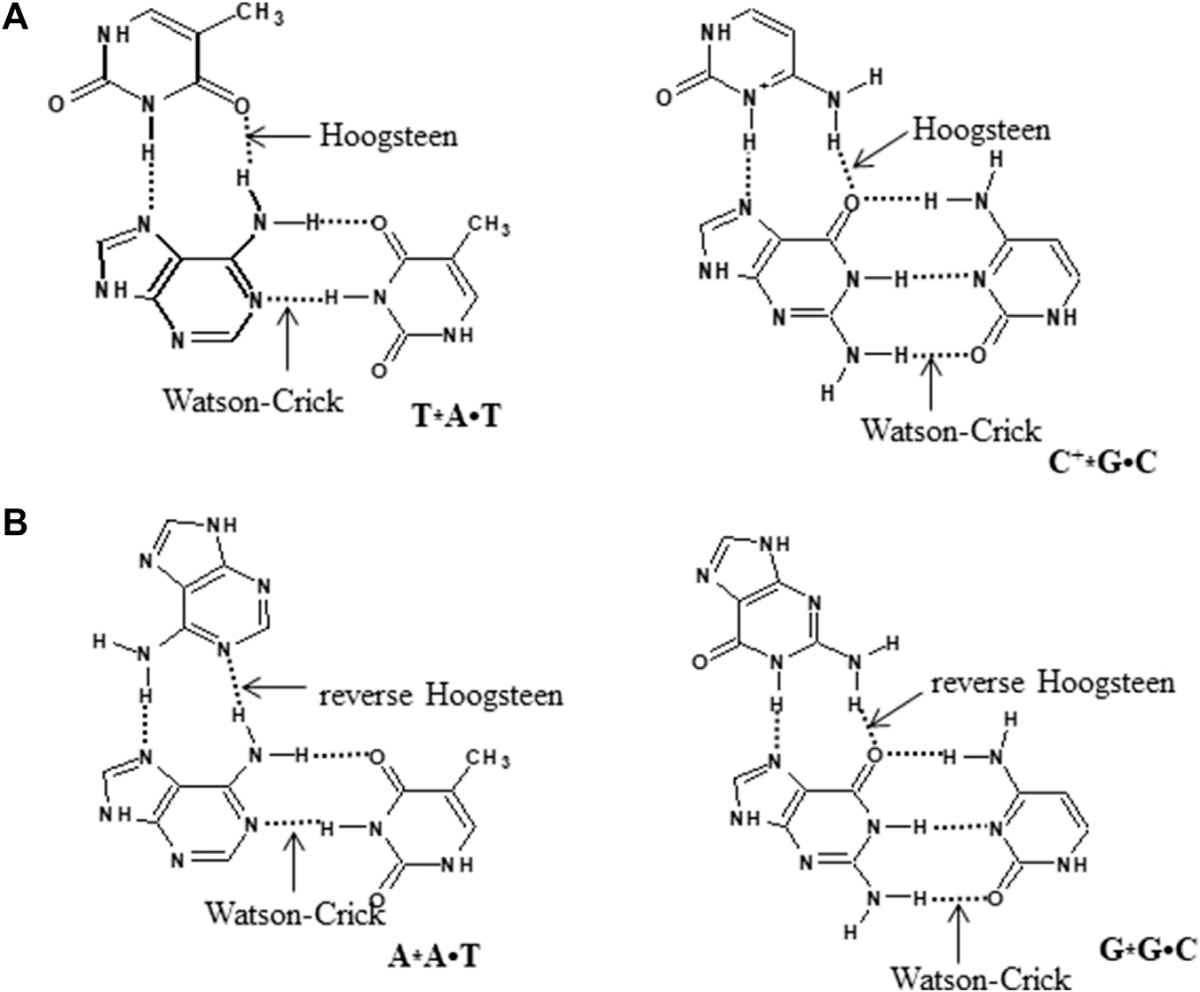
Hydrogen bonding pattern involved in DNA triplexes **(A)** pyrimidine motif and **(B)** purine motif.

**FIGURE 8:**
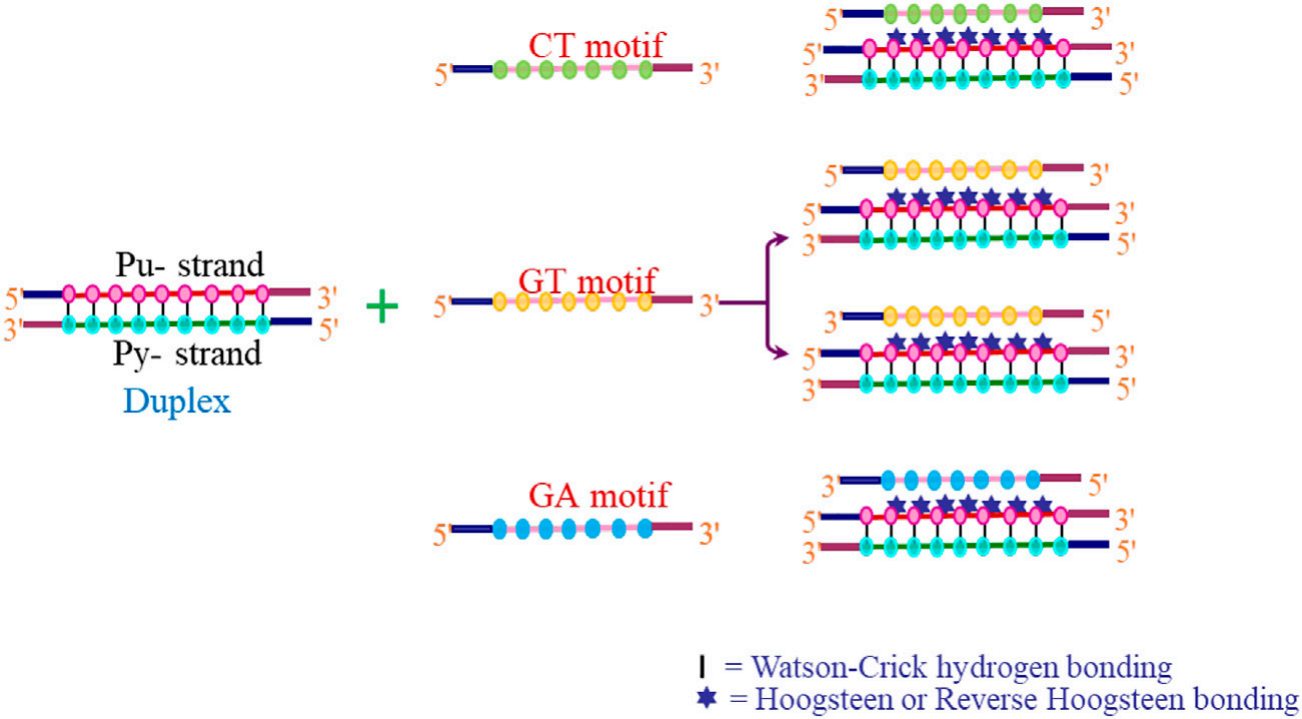
Possibilities of intermolecular triplex formation.

The interaction of a third strand to the major groove of the duplex DNA in a sequence-specific manner results in the formation of an intermolecular triplex, and these structures have been shown to exhibit immense potential as transcription inhibition agents and site-directed mutagens. Depending on the relative orientation and base composition of the third strand (TFO), triple helices can be broadly categorized into three categories. In pyrimidine motif (Py triplexes), the triplex-forming oligonucleotide (TFO) that binds to the target duplex consists of cytosine and thymine (C, T) and have parallel orientation to the central purine-rich strand of duplex DNA. The third strand interact with duplex *via* Hoogsteen hydrogen bonds and form T•A*T and C•G*C^+^ base triplets (• represents Watson–Crick hydrogen bonds and * depicts Hoogsteen hydrogen bonds). These triplexes are particularly stable at acidic pH, which facilitates protonation of cytosine at N3 position (Husler and Klump 1995; Asensio et al., 1998) (Figure 7). The formation of intermolecular pyrimidine motif triplex by a homopurine and homopyrimidine DNA strand at 1:1 ratio and low pH by nuclear magnetic resonance (NMR) has been demonstrated by Bhaumik et al. (1995). Another interesting work on similar lines demonstrated the formation of a novel palindromic triple-stranded structure by d-CTTCTCCTCTTC and d-GAAGAG. They used NMR and molecular dynamics for the structure elucidation, and also discussed the effect of hydrogen ion concentration and temperature on triplex stability (Bhaumik et al., 1998). Since intermolecular pyrimidine motif triplexes are unstable at physiological pH, various approaches are employed to stabilize these structures (Kukreti et al., 1997, 1998). However, low pH is not a condition for intramolecular pyrimidine motif triplex formation, and such structures are found to be stable even at neutral pH (Plum and Breslauer 1995; Kaushik and Kukreti 2021). Purine motif triplexes are formed when a TFO consisting of guanines and adenines (G, A ODNs) interacts with the polypurine strand of target duplex in an antiparallel manner *via* reverse Hoogsteen interactions forming T•A*A and C•G*G base triplets. These structures are formed at or near physiological pH. Intermolecular purine motif triplex formation depends on divalent cations such as magnesium (Pilch et al., 1991; Frank-Kamenetskii and Mirkin 1995; Francois et al., 2000); however, their intramolecular counterparts can form even in the absence of divalent cations (Kaushik et al., 2011). In the third category, TFO consisting of thymines and guanines (T, G) interact *via* either Hoogsteen/reverse Hoogsteen bonding, and align in parallel or antiparallel manner to the purine strand of DNA duplex respectively to form a triplex (Figure 8).

Sequence considerations play an important role in determining the relative stability of DNA triplexes, especially under physiological conditions. These structures exhibit numerous biological applications, for instance they are involved in replication pausing, regulation of gene expression, manipulating gene silencing, gene-targeted mutagenesis, cell proliferation, and inhibition of virus propagation through triple-helix formation (Helene 1991; Praseuth et al., 1999; Krasilnikova and Mirkin 2004; Mukherjee and Vasquez 2011).

### DNA triplexes and associated diseases

**FIGURE 9:**
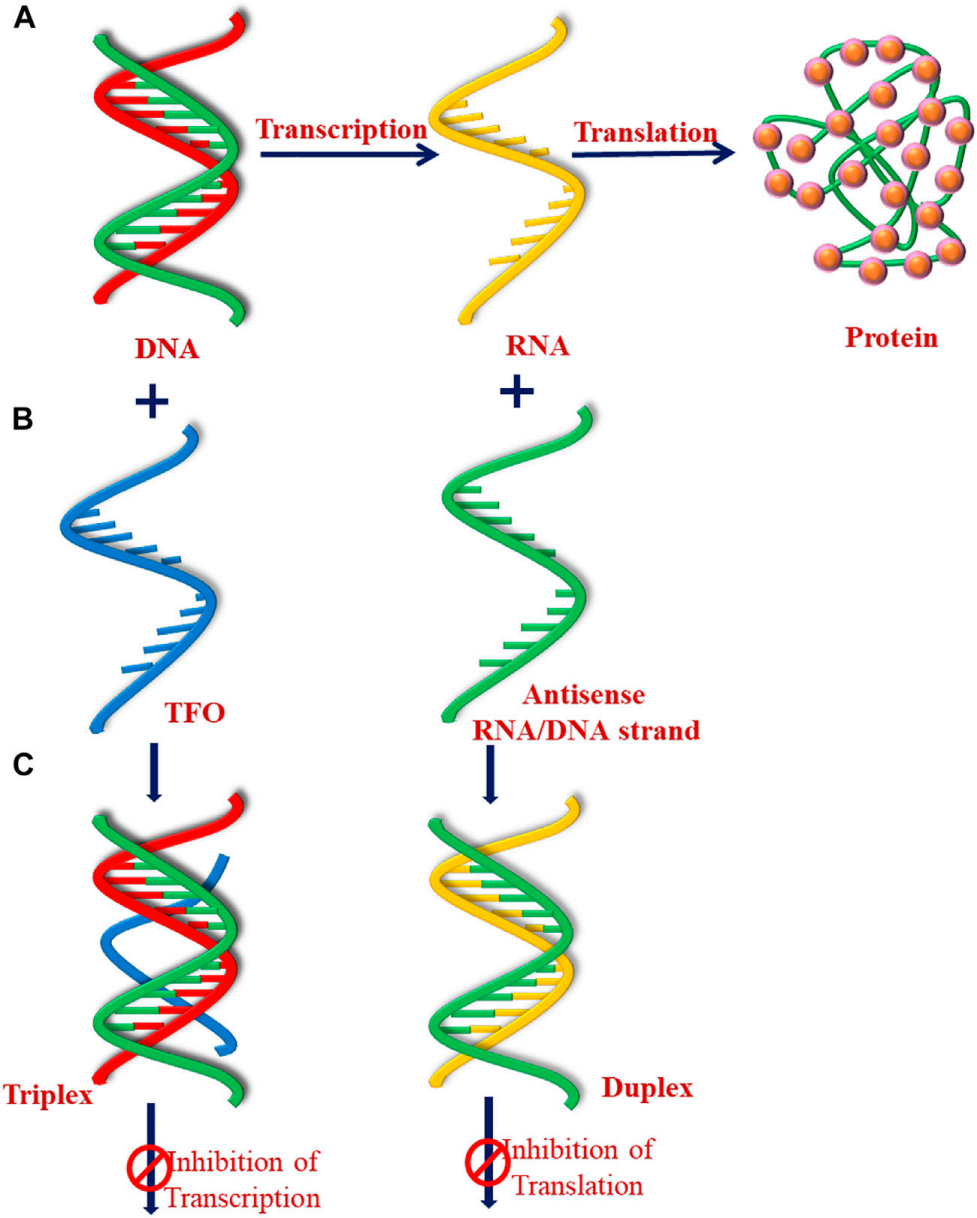
Schematic represent of **(A)** central dogma of molecular biology, **(B)** antigene, and **(C)** antisense strategy.

Under normal cellular conditions, the number of cells in an organism remains constant *via* cell division and proliferation. These are highly regulated processes in normal healthy tissue; however, genetic mutations can transform a healthy cell into diseased cancer cells. According to the “central dogma of molecular biology” (Figure 9), the genetic information stored in DNA duplex is first transcribed to messenger RNA (mRNA), and later transferred to the proteins *via* the process of translation. In general, gene expression is considered to be regulated by proteins such as transcription factors; however, genetic mutations interfere with these biological processes and affect protein production. Understanding the fundamental mechanisms involved in transcription and translation is of utmost medical importance because interference at this molecular level may lead to various human ailments such as cancer, heart diseases, neurological diseases, and various types of inflammation. Various environmental factors such as exposure to UV radiations and air pollutants, poor lifestyle (high alcohol intake, smoking, and tobacco use), and certain microbial infections have also been reported to induce genetic mutations, and damage DNA (Sasco et al., 2004; Hopkinson et al., 2006).

**FIGURE 10:**
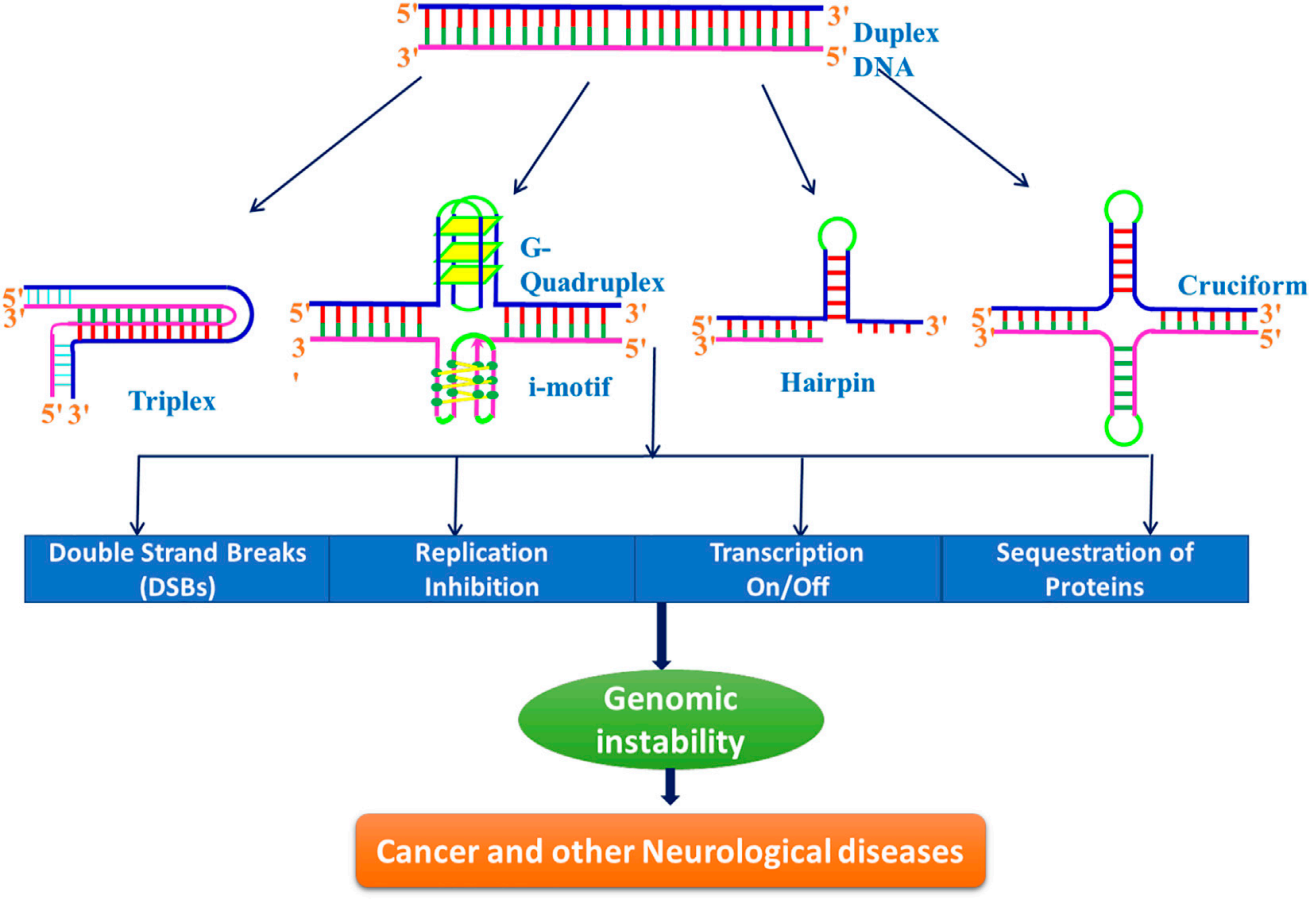
Schematic representation of role of different non-B-DNA structures in inducing genomic instability.

Human genomes are rich in repetitive DNA sequences, known as short tandem repeats (STRs) or microsatellites. Although, larger repeats (tetranucleotides and pentanucleotides) also exist in human genome; the widely studied sequences are trinucleotide repeats (TNRs) such as CGG, CAG, CTG, GAA, CCG, and CUG found, both in the coding and non-coding regions. Being polymorphic, they are more susceptible to alternations during various biological processes, and hence widely used in biological research (Fan and Chu 2007). The unstable microsatellite repeats have been identified as the cause of hereditary neurological disorders in humans in 1991. This discovery marked a turning point in the field of non-canonical DNA structures and firmly established that the formation of non-B-DNA structures in living cells induces genetic instability, leading to many human diseases (Figure 10) (Well et al., 1998; Bacolla and Wells 2004; Wang and Vasquez 2004). Trinucleotide repeat (TNR) is known to cause many rare, dominant, and neurological disorders such as Huntington’s disease, Friedreich ataxia, fragile X syndrome, myotonic dystrophy, and certain types of spinocerebellar ataxia. A total of 47 STR disease-causing genes have been reported to date. This list of neurological disorders associated with the expansion of repetitive sequences is growing rapidly, and recent addition to this is RFC1, VWA1, GIPC1, NOTCH2NLC, and LRP12 genes (Chintalaphani et al., 2021). Numerous potential nucleic acid tools such as triplex-forming oligonucleotides (TFOs), antisense oligonucleotides (ASOs), microRNAs (miRNAs), small interfering RNAs (siRNA), DNAzymes, aptamers, and immunoregulatory oligonucleotides have been used to treat cancer and other neurological diseases (Meng et al., 2016; Bennett et al., 2019; Lin et al., 2020; Oh et al., 2020; Hill and Meisler, 2021; Wu et al., 2022). All of these are small oligonucleotides that can bind to specific sequences in the DNA, RNA, or proteins, and therefore, can inhibit transcription (antigene strategy), translation (antisense strategy), splicing, or protein activity.

The biological role of DNA triplex has been a mystery since its discovery in the late 1950s. Polypurine•polypyrimidine tracts are widespread in the human and other genomes, and these sites represent the potential to cause as well as a therapeutic target to treat many diseases. Several reports on triple-helical structures have shown a direct connection between these structures and various inherited as well as acquired human diseases, including cancer and other neurological disorders like FRDA, fragile X syndrome, muscular dystrophy, and spinocerebellar ataxia (Pollard et al., 2004; Raghavan et al., 2005; Wang and Vasquez, 2006, 2009; Liu et al., 2012). During the replication process, DNA double-strand must be unwound to a single-stranded state, and this phenomenon increases the possibility of the formation of various non-canonical DNA structures, which can affect replication. Replication errors cause mutations and recombination of genomic DNA, causing various serious diseases. Ample evidence have demonstrated the involvement of triplexes in mutagenesis, genetic instabilities, and DNA repair and recombination. The scientific findings have also indicated the role of DNA triplexes in a wide array of genetic rearrangements, such as deletions, translocations, inversion, insertion, and duplications. A new paradigm in disease etiology revealed rearrangements as the genetic basis of approximately 50 human diseases, including follicular lymphomas, adrenoleukodystrophy, and spermatogenic failure. Studies have shown that the formation of a DNA triplex at lagging strand during replication results in fork collapse (Frick and Richardson 2001) and induce genomic instability *via* blocking the DNA replication and transcription elongation (Bétous et al., 2009; Liu et al., 2012). These structures have also been shown to involve in disease onset and progression. For example, triplex formation in the promotor region of c-myc oncogene hampers the process of transcription (Belotserkovskii et al., 2007), and c-myc translocations are often associated with leukemias and lymphomas (Haluska et al., 1988; Joos et al., 1992b; Saglio et al., 1993; Wang and Vasquez 2004). Burkitt’s lymphoma in which t [8; 14] translocation occurs is also associated with triplex formation (Table 2).

**TABLE 2:** Human genetic diseases associated with DNA triplexes.

Disease	Type of cell	Affected gene	Function	Reference
Autosomal dominant polycystic kidney disease (ADPKD)	Renal (kidney) cells	PKD1 and PKD2	Formation of polycystins	Van Raay et al. 1996; Bagheri et al. 2019
Friedreich’s ataxia	COS-7	Frataxin gene	Regulating cellular iron homeostasis	Ohshima et al. (1998)
Cancer (Burkitt’s lymphoma)	BL cell lines	c-myc	Regulating the transcription	Joos et al. 1992a; Wang and Vasquez 2004
Cancer (breast and ovarian cancer)	HeLa cells (cervical cancer cells)	BRCA1- associated proteins	Regulating the transcription	Wang et al. (2000)

### Autosomal dominant polycystic kidney disease (ADPKD)

ADPKD, one of the most common genetic disorders across the world is characterized by the formation of cysts within the kidneys (Dalgaard 1957). It affects approximately 1:800 individuals, and in the United States, this is responsible for up to 10% of cases of end-stage renal disease (Gabow 1993). ADPKD is not simply a kidney disease but other organ systems (multisystem disorder) also get affected by the formation of cysts. Cardiovascular impediments such as aortic aneurysms and valvular abnormalities have also been observed in an individual (Torres et al., 2007; Bansal et al., 2019).

Genetic analysis revealed that ADPKD is caused by mutations in one of two protein-coding genes, polycystic kidney disease 1, PKD1 [16p13.3], or PKD2 [4q21-23]. These genes make novel proteins, polycystins, which are essential for the proper health of the kidneys and other parts of the body. PKD1 is associated with more than 85% of the ADPKD cases, which is the most aggressive form of the disease; whereas PKD2 is responsible for 15% of patients who are found to be mutation positive (Rossetti et al., 2012).

The human PKD1 gene contains two long stretches of polypyrimidine tracts in intron-21 (2.5 kb) and -22 (602 bp), and the PKD1 intron-21 Pu•Py tract contains 97% pyrimidine (65% cytosine and 32% thymine) on the coding strand. This is one of the largest intragenic Pu•Py tracts found in the human genome that contain 23 mirror repeat sequences. Blaszak et al. (1999) used primer extension reactions with chloroacetaldehyde and potassium permanganate modification to show the formation of triplex DNA within this Pu•Py tract. Using the same tract, Tiner et al. (2001) have shown the formation of intramolecular triplex by atomic force microscopy. The effect of PKD1 Pu•Py tracts on eukaryotic systems has been studied by Patel et al. (2004) using primer extension assay and an SV40 system. Results revealed that this sequence form an intramolecular DNA triplex and strongly interferes with DNA replication in the eukaryotic system. They have also demonstrated that there exists a correlation between replication blockade, and the potential length of the triplex as well as the superhelical tension of intramolecular triplex formation. Bacolla et al. (2001) have also studied the stability of the same tract in a bacterial model and have shown that this sequence causes a mutation in the PKD1 gene by stimulating repair and/or recombination functions. The inhibition of replication, generation of double-strand breaks, and rounds of DNA repair/reactivation of DNA forks are responsible for the generation of a high new germline mutation rate in the PKD1 gene (Bissler 2007). All these findings have suggested the role of Pu•Py tracts in facilitating somatic mutations that lead to the development of ADPKD.

#### Friedreich’s ataxia

Friedreich’s ataxia [FRDA] is an autosomal recessive neurodegenerative disorder that affects peripheral nerves, the spinal cord, and the cerebellum portion of the brain. It is one of the most common forms of autosomal recessive ataxia that affects about 1 in every 50,000 individuals. FRDA is characterized by muscle weakness, spasticity in the lower limbs, bladder dysfunction, scoliosis, absent lower limb reflexes, and loss of position and vibration sense (Bidichandani and Delatycki 1993). This condition is named after German doctor Nikolaus Friedreich, who first described this disease in 1863. Friedreich ataxia is the first known example of an autosomal recessive genetic disease caused by the massive expansion of triplet [GAA] repeat. Mutation of FRDA gene called FXN [or X25], which is present on chromosome 9q21 has been identified to cause FRDA. FXN contains seven exons and encodes a mitochondrial protein, frataxin which plays an important role in regulating cellular iron homeostasis by binding iron (Rajeshwari 2012). The purine strand present in the expanded repeat region of the DNA folds back to form purine motif triplex (Py•Pu*Pu), which inhibits the gene expression of frataxin.

The expansion of the GAA•TTC tract in intron 1 leads to the transcriptional silencing, and the plausible mechanisms are [i] the formation of triplexes [Sticky DNA], which stalls RNA polymerase, [ii] the creation of a persistent DNA–RNA hybrid, or [iii] heterochromatin formation (Wells 2008). Delatycki et al. (1999) stated that the expansion of a GAA trinucleotide repeat sequence (TRS) in the first intron of the FXN gene is responsible for approximately 98% of FRDA cases, and the other 2% occurs due to point mutations in the FXN gene. A study on similar lines also indicated that 97% of cases of Friedreich ataxia are linked to GAA triplet repeat expansion (Lodi et al., 1999).

#### Follicular lymphoma

Analyses of the DNA sequences have revealed that the formation of some alternative DNA structures adversely affects DNA replication, repair, and recombination, and may lead to mutations. There are various pathways by which DNA can repair itself, and if not repaired, double-strand breaks (DSBs) can lead to cell death. Even in case, it is not repaired correctly, DSBs can potentiate chromosomal translocations, deletions, and fusion in the DNA.

The most common chromosomal translocation in human cancer is t [14; 18][q32; q21], and this translocation occurs in almost all follicular lymphomas. In most follicular lymphoma carriers, the break at chromosome 18 occurs within a small 150-bp region, termed as a major breakpoint region [Mbr], and three translocation hot spots have been identified within this 150-bp Mbr region. On the basis of gel assays, electron microscopy, and circular dichroism, Raghavan et al. (2005) have shown the formation of triplex DNA at the bcl-2 Mbr under physiological conditions, and this non-B-DNA structure within the bcl-2 Mbr leads to t[14;18] translocation.

It has already been discussed that sequences capable of forming alternative DNA structures induce genetic instability that resulted in generating mutagenic hot spots in the genome, and one such example is c-myc gene. c-myc is one of the most important transcriptional factors that plays a crucial role in regulating diverse array of cellular pathways (Nguyen et al., 2017). Chromosomal translocation t[8,14] of the c-myc gene to an immunoglobulin gene resulted in the development of Burkitt’s lymphoma [BL]. In BL, translocation that involves the movement of part of chromosome 8 containing the c-myc gene onto the immunoglobulin [Ig] heavy chain gene locus on chromosome 14 [14q32], is the most common, occurs in approximately 80% of cases, while a significant minority of translocations also involves the immunoglobin light chain genes at chromosome 2 [κ][2p12] or 22 [λ][22q11]. Previous studies have well-illustrated the connection between the formation of non-B-DNA structures within the c-myc promoter region and chromosomal translocation in the Burkett lymphoma (Wang and Vasquez 2004; Wilda et al., 2004; Del Mundo et al., 2017). The formation of antiparallel purine motif triplex in the c-myc promoter region has been shown by Umek and coworkers using electrophoretic mobility shift assays. They have also studied the formation and influence of an intramolecular [H-DNA] triplex on the strand invading capability of antigene LNA-ONs [locked nucleic acid-oligonucleotides] and suggested that the formation of such structures facilitates dsDNA strand-invasion of the antigene ONs (Umek et al., 2019). A study on similar lines has also shown that the formation of a triplex structure at the human c-myc promoter interferes with the transcription process (Belotserkovskii et al., 2007). They have also suggested the various plausible mechanisms of DNA triplex-induced transcription inhibition and potential biological implications.

### DNA triple-helical structures as therapeutic tools

Gene products play a crucial role in regulating various cellular processes such as cell signaling, transcription, translation, and oncogenesis. Triplex formation targeting nucleic acids provides an interesting therapeutic approach that can be employed to control the processes involved in disease onset and progression. Two important mechanisms can prevent the progression of tumors in the normal cells, [i] activation of the tumor suppressor (TS) gene and [ii] inactivation of a proto-oncogene. Various genes such as brca1, p53, and Rb encode tumor suppressor proteins, which suppress tumor formation. Mutation in the TS gene or abnormal expression of TS proteins leads to oncogenesis. Also, an alteration (mutation) in the proto-oncogene results in an abnormality in the structure and function of a protein that can transform a normal cell into cancer cells. The relationship between non-canonical DNA structures such as triplexes and oncogenesis has been widely studied in recent years.

**FIGURE 11:**
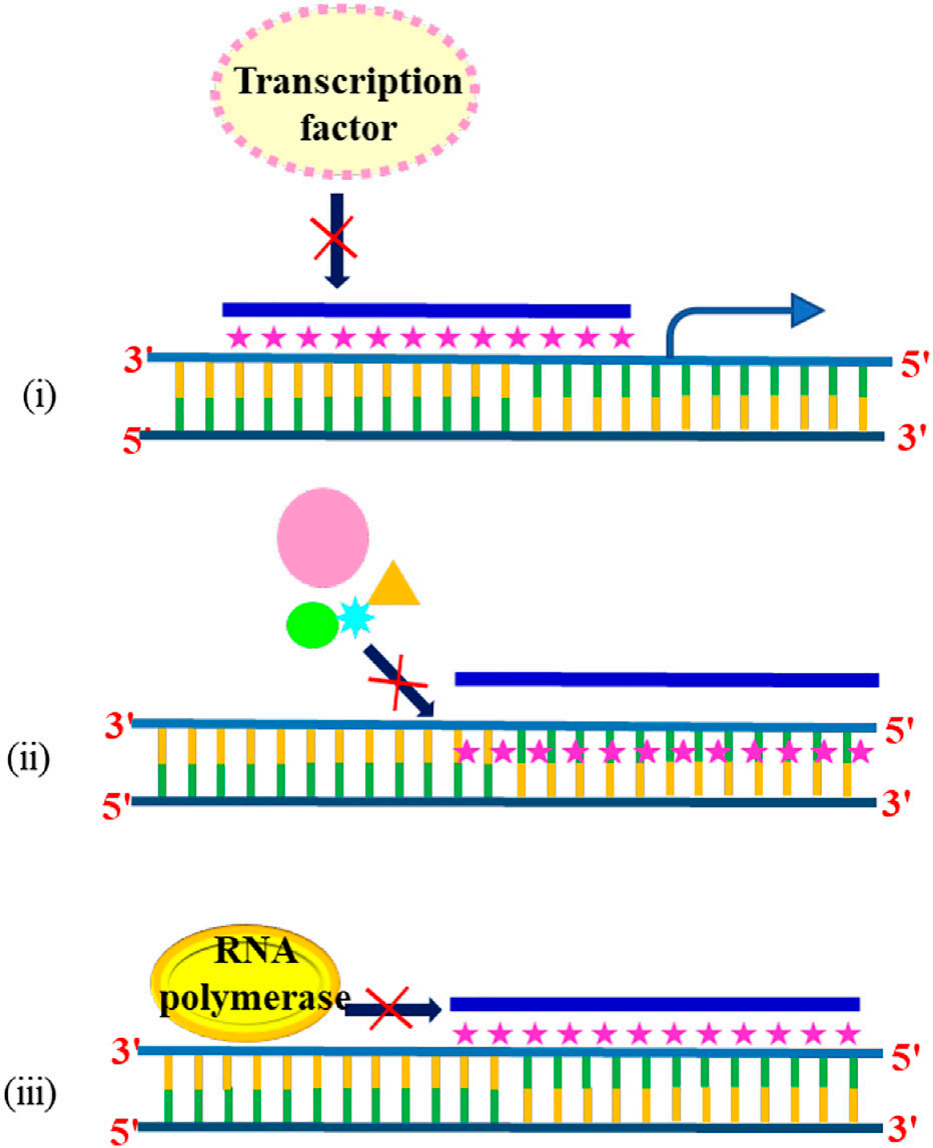
Triplex formation inhibits the gene expression by (i) promoter occlusion, (ii) inhibiting transcription initiation, and (iii) blocking RNA polymerase.

Triplex-specific effects have been the subject of intense research among scientists, and several reports have revealed the effect of triplex DNA on the transcription of cancer-causing genes. The formation of DNA triplex structures inhibits the gene expression by any of the mechanisms: [i] promoter occlusion, also called transcriptional interference—where triplex formation at the promoter site does not allow transcription factors to bind (Mcshan 1992), [ii] by inhibiting transcription initiation (Duval-Valentin et al., 1992), or [iii] directly blocking progression of RNA polymerase (Scaggiante 1994) as shown in Figure 11. The first example of TFO-directed inhibition of gene expression was demonstrated by Postel and others in 1991. They have shown that triplex formation at the c-myc gene in HeLa cells reduced the c-myc expression, and the inhibition was found to be site as well as sequence-specific (Postel et al., 1991). Following this pioneering work, the effects of TFOs in transcription were studied on many genes, including Ha-ras, the HER-2/neu/c-erbB2 proto-oncogene, cyclin D1, and the alpha subunit of the interleukin-2 receptor (Ebbinghaus et al., 1993; Grigoriev et al., 1993; Kim and Miller, 1998).

The high-mobility group box 1 (hmgb1) gene encodes a protein that plays a crucial role in several cellular processes, and aberrant expression of hmgb1 has been reported to cause cancer. The selective transcriptional inhibition of the hmgb1 gene using triplex-based gene technology has been studied in a recent work (Lohani and Rajeshwari 2020). We have recently reported the formation of an intermolecular (Py-motif) triplex at 27-bp genomic homopurine–homopyrimidine tract present in the human DACH1 gene (Khan et al., 2021). This gene encodes for a chromatin-associated protein, DACH1, and expression of this gene is linked with various diseases including cancer. This work suggested that targeting the gene *via* triplex formation can help in elucidating the tissue-specific physiological function and will enhance the understanding of the disease pathophysiology from inhibition of DACH1 expression.

In addition to binding at the promoter site, another mechanism to hamper the prolongation of transcription is by blocking the progression of the RNA polymerase. Krasilnikov et al. (1997) have emphasized that there are fundamental differences in the way DNA polymerase responds to DNA triplex formation representing a barrier to polymerization, and it is attributed to the inability of polymerase to dissociate triplex structure. This work demonstrated that significantly higher temperatures were required to dissociate intramolecular triplexes than the corresponding duplex sequences, which leads to DNA polymerase arrest during replication. Another study on similar lines employed N3′-P5′ phosphoramidate (np)-modified TFO to target a polypurine tract (PPT) in the coding region of two HIV-1 genes, revealed a 40–50% decrease in reporter RNA expression in both, transient and stable expression systems, implying the significance of specific polymerase arrest (Faria et al., 2000). DNA triplex-based therapeutic approach has also revealed remarkable results in the treatment of malignancies associated with MET (mesenchymal epithelial transition factor) overexpression (Singhal 2011). These studies clearly described the importance of triplex-based strategies for specific polymerase arrest, thus inhibiting gene expression.

Another well-studied and potential therapeutic application of triplex technology is the use of TFOs to target site-specific mutations in the genome to correct/inactivate the target gene. Naturally occurring H-DNA-forming sequences have been shown to induce mutagenesis in cells and in mice, suggesting the mutagenic potential of triplex-forming structures *in vivo*. Studies have also revealed that TFOs can effectively induce targeted mutagenesis on chromosomal DNA in mammalian cells (Vasquez et al., 1999, 2000). Triplex-based gene therapy is also used as an effective approach for the treatment of autosomal dominant disorders, and the action of mutant/deleterious genes can be inactivated or corrected by stimulating recombination. Autosomal dominant retinitis pigmentosa (ADRP), a rare, inherited degenerative disease, is caused by deleterious protein expression that affects the photoreceptor cells of the retina, causing severe vision impairment, and progressive retinal degeneration ultimately leading to blindness. Although several genes associated with mutations in the RHO gene, encoding a G protein-coupled receptor involved in the manifestation of blindness, are the most common cause of ADRP (Dryja and Li 1995). Triplex-mediated DNA photo-crosslinking has been used for blocking the transcription of the human rhodopsin gene (Intody et al., 2000). Results demonstrated that directing DNA damage with psoralen-TFOs is an efficient and specific means of inhibiting the gene expression.

Homologous recombination (HR) plays an important role in DNA repair, replication, and several other aspects of chromosome maintenance. Among other strategies, sequence-specific DNA repair by TFOs is considered a promising tool to enhance recombinogenic frequencies. The recombinogenic potential of TFOs and TFO-directed psoralen inter-strand crosslinks [ICLS] in intermolecular, intramolecular, and the chromosomal context in mammalian cells has been studied by various researchers (Vasquez et al., 2001; Knauert 2006; Liu et al., 2008). Faruqi et al. (2000) have shown intermolecular triple-helix-induced homologous recombination in mammalian cells using a simian virus SV40-based shuttle vector, and the third-strand stimulated recombination was found to be dependent on the nucleotide excision repair [NER] pathway .

Although, triple-helical structures are formed with precise sequence specificity; their formation is a thermodynamically weaker and kinetically slower phenomenon than duplex formation. The inherent instability of triplexes has resulted in the development of various triplex-specific ligands, and TFOs have also been modified by attaching various intercalating agents to enhance triplex stability. The presence of divalent cations such as Mg^2+^ and Ca^2+^ as well as polyamines such as spermidine, spermine, and putrescine also stabilize these structures.

The literature is rich in reports illustrating the stabilization of DNA triplexes with intercalators, and majority of these ligands were evaluated on intermolecular triplexes; however, such molecules have a profound effect on intramolecular triplexes as well. B [e]PI was the first molecule reported by Claude Helene’s group, which preferentially and strongly stabilizes intermolecular triplexes rather than the underlying duplexes. It selectively stabilizes regions of T•A*T rather than C•G*C^+^ because a positive charge on protonated cytosine prevents its binding (Mergny et al., 1992). Existing studies have well demonstrated that targeting nucleic acid triplexes with small molecules has implications in the therapeutic and diagnostic field for the treatment of numerous diseases such as lupus, diabetes, hemophilia, Huntington’s disease, and many other genetic disorders. Thus, triplex-related interactions using small molecules present a promising gene targeting strategy, well-reviewed by Fox (2000).

## G-quadruplex structures

**FIGURE 12:**
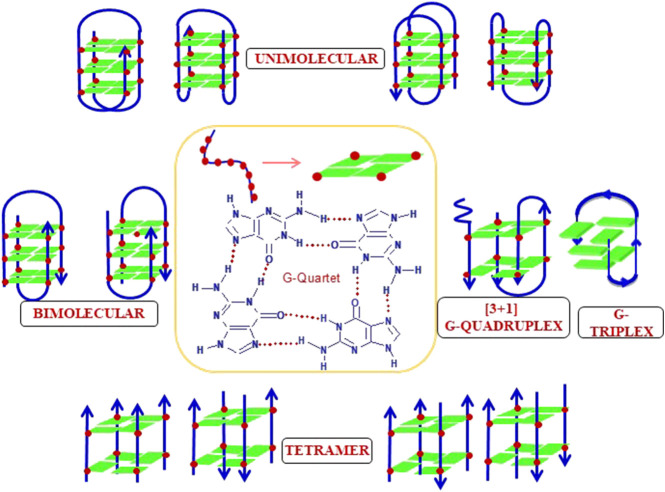
Different topologies adopted by a G-quadruplex structure.

The structural dynamics of DNA make it capable of adopting alternate non-B-DNA structures. Among all the five nucleosides found in DNA and RNA, guanosine is the only one capable of undergoing self-association in the solution to form G·G base pairs using Hoogsteen hydrogen bonding and thus form higher order non-canonical structures known as G-quartet/G-quadruplex as shown in Figure 12. The G-quadruplexes are very well known for their polymorphic behavior due to inter- or intramolecular folding of G-rich strands (Burge et al., 2006; Patel et al., 2007; Neidle 2009; Phan 2010; Agarwala et al., 2015; Bansal and Kukreti 2020). Also, many factors like the number of stacked G-quartet, strand orientation, loop length, and presence of counterions, which contribute to the formation and stabilization of G-quadruplexes. Multiple studies have delineated the rules to predict the stability of different polymorphic forms of G-quadruplex with different repeats, loop length, presence of ions *etc*. Using different techniques like circular dichroism, gel electrophoresis, and thermal melting, Rachwal and coworkers explained the stability of intramolecular G-quadruplex formed by the oligonucleotide sequence of the type d(GnT)_4_ and d(GnT_2_)_4_, where *n* = 3–7 in the presence of Na^+^ and K^+^. They revealed the formation of intramolecular G-quadruplex with both the sequences. Their stability increases with increase in the sequence length (*n* = 7 > *n* = 6 > *n* = 5 and so on) in case of d(GnT_2_)_4_, but the trend was not regular in case of d (GnT)_4_. The major conformation depicted in case of K^+^ was found to be parallel while the presence of Na^+^ favors antiparallel conformation (Rachwal et al., 2007). Furthermore, the role of loop length in the folding pattern of G-quadruplex has been demonstrated by Hezel et al. They showed that in the presence of single residue loop, the only possibility for intramolecular G-quadruplexes is to fold into parallel conformation. When loop length is more (>2 residue) or single thymine with other loop residues, the G-quadruplexes have the potential to fold into both parallel and antiparallel topology. Also, the loop length contributes in determining the stability of G-quadruplexes. They also demonstrated the melting studies of various nucleotide sequences with different loop length and unveil that short loop length shows more stability as they are energetically more stable (Hazel et al., 2004). The structure and stability of the polymorphic forms of G-quadruplexes have always been the area to explore since after its discovery. Numerous studies have documented in the literature unveiling about structural heterogeneity of G-quadruplexes under different parameters (reviewed by Dolinnaya et al., 2016), like, sequence length, loop length, and cations also determine the stability and the folding of G-quadruplex. The interaction between the ions and G-quadruplex is based on their arrangement along the G-quartet plane or between the planes depending on the size, charge, interactions with the loop, groove *etc*, and thus determine the stability and the structure of the G-quadruplex (reviewed by Bhattacharyya et al., 2016). Maximum studies have involved Na^+^ and K^+^ as these two are the most physiologically relevant ions, but the role of some divalent ions have also been examined. In an early study by Hardin et al. (1992), the order of ions in determining the stability of cations was suggested as K^+^ > Ca^2+^ > Na^+^ > Mg^2+^ > Li^+^ and K^+^ > Rb^+^ > Cs^+^. Furthermore, study by Vanczel and Sen 1993, the order of the ions from IA and IIA were given as Sr^2+^ > Ba^2+^> Ca^2+^ > Mg^2+^ and K^+^ > Rb^+^ > Na^+^ > Li^+^ = Cs^+^.

Another very important factor which decides the mode of interaction of any ion to G-quartet is its ionic radius and hydration energy. For example, K^+^ and NH^4+^ have ionic radii 1.33 and 1.48 Å, respectively, which is very large to fit within the plane of a G-quartet, while Na^+^ is very small to move within the plane (Laughlan et al., 1994). Moreover, the hydration energy is inversely related to ionic radii. It is reported that free energy for binding Na^+^ is more favorable than that of K^+^ but the energy required to coordinate K^+^ within G-quadruplex is far less than the energy required to dehydrate Na^+^. Thus, the net difference between the two energies determines the cation selectivity for G-quadruplex. Hence, higher energetic cost of Na^+^ in comparison to K^+^, makes K^+^ more preferred for G-quadruplex binding (Gu et al., 2002; Sacca et al., 2005).

The elegant studies by Hosur’s group in deciphering the structural diversity of G-quadruplexes came out with some novel structures like A-tetrad and T-tetrad (Patel and Hosur 1999; Patel, Koti and Hosur 1999). It has been demonstrated that double repeat (d-TGGTGGC) of *Saccharomyces cerevisiae* telomere forms a parallel quadruplex, comprising both G-tetrad and T-tetrad in the presence of K^+^ ions. This observation indicated that like guanine, thymine also carries the potential to adopt diverse structures. The presence of single T can be adjusted easily without disturbing the structure, while presence of more than one T can be arranged only in the formation of loop in a G-quadruplex. Likewise, presence of single adenine (A) in the two truncated human telomere sequence, d-AG_3_T and d-TAG_3_T were studied in K^+^ and revealed different structural arrangements. It has been shown that both the sequences form parallel G-quadruplexes; however, an additional interesting feature of A-tetrad formation at 5′-end was also observed in d-AG_3_T and not in the d-TAG_3_T (Patel and Hosur 1999; Patel, Koti and Hosur 1999).

Kettani et al. (1998) came forth with a new sequence-specific structure formation demonstrated in a dodecanucleotide sequenced (G-G-G-C-T_4_-G-G-G-C) found in adeno-associated human parvovirus. The studied dodecamer was found to form an antiparallel quadruplex in the presence of Na^+^, with two G-tetrad flanked by GCGC tetrad connecting T_4_ lateral loop (Kettani et al., 1998). Similarly, another sequence from human telomeric variant with four CTAGGG repeats was shown to form a novel antiparallel G-quadruplex with two G-tetrads sandwiched between G•C•G•C tetrad and G•C base pair (Lim et al., 2009).

Mounting evidence of presence of contiguous stretches of G’s are found not only in the telomeric region of eukaryotic chromosomes but also in other genomic locations (Sen and Gilbert 1988; Fry and Loeb 1994; SanthanaMariappan et al., 1996), promoter regions of several human proto-oncogenes including retinoblastoma gene susceptibility (Murchie and Lilley 1992) and c-myc (Simonsson et al., 1998), mutation and recombination hot spots (Simonsson 2001), and UTR’s (untranslated region) (Kumari et al., 2007). Moreover, using the high resolution sequencing based method, the occurrence and the distribution of these G4 motifs have been reported in the human genome by Chambers group. Based on mounting evidence, they also reported the association of G-quadruplex with oncogenes, somatic genes related to cancer development, and thus represent a promising approach for cancer intervention (Chambers et al., 2015). In another interesting study by Schaffitzal et al., the actual existence of G-quadruplex in the nucleus was experimented by high-affinity binder taken from human combinatorial antibody library. Several single-chain antibody fragments (ScFv) were taken to detect their binding, especially with G-quadruplex formed by *Stylonychia* telomeric repeats. It has been found that one of the ScFv selected (Sty3) had affinity for parallel G-qudruplex 1,000 times more than antiparallel conformation, while another fragment Sty49 showed the same affinity for both parallel and antiparallel quadruplex (Schaffitzal et al., 2001). Their landmark studies also revealed the importance of G-quadruplex in the telomere functioning as no staining of the replication band was found in the interaction of macronuclei of *Stylonychia* telomere with Sty49, which proved G-quadruplex resolved during replication (Schaffitzal et al., 2001). An elegant review by Datta et al. has nicely described the G-quadruplex biology in ribosomal DNA (rDNA). It has been demonstrated that G4 targeting in ribosomes and other loci play a vital role in regulation of biological processes and genomic stability and also provide a useful approach to combat with cancer (Datta et al., 2021).

Thus, G-quadruplexes has emerged as a potential therapeutic insinuation due to their potential to adopt diverse topologies, and various biological functions like gene regulation, DNA replication, transcription, translation, telomere maintenance (Siddiqui-Jain et al., 2002; Phan et al., 2005; Gunaratnam et al., 2009; Verma et al., 2009; Brooks et al., 2010; Brosh 2013), and therapeutic targets (Balasubramanian and Neidle 2009; Cosconati et al., 2010; Rhodes and Lipps 2015). G-quadruplexes found near the origin of the replication, and may regulate its initiation. Furthermore, replication of DNA G-motifs require the unfolding of DNA by helicases, an important class of proteins that play an important role to correct endogenous- or exogenous-induced DNA damage or replication error. In the absence of or in deficiency of these helicases-dependent pathways, the genomic integrity is compromised, which results in mutagenesis, cellular senescence and death, carcinogenesis, and neurological disease. Mutation in helicases acting on G-quadruplex structures (BLM, WRN, and FANCJ) cause genetic instability.

### Diseases associated with replication helicases involving G4-motifs

1. α-thalassemia intellectual disability syndrome is a genetic condition characterized by intellectual disability, muscle weakness, short height, *etc*. It is caused by the mutation in ATRX protein (ATP dependent helicase) encoded by ATRX gene, which is common in tumors of the central nervous system. It activates the maintenance of telomere by the telomerase-independent ALT (alternate lengthening telomere) pathway. The ATRX is found at telomeres where their target is a variable number of tandem repeats of G-rich sequences found at α-globin gene (CGCGGGGCGGGGG)n, which forms G-quadruplexes. The absence or the deficiency of ATRX causes downregulating α-globin gene expression and causes α-thalassemia (Law et al., 2010; Sarkies et al., 2010; Shay et al., 2012)
2. Werner syndrome is caused by mutation of RecQ3 helicase (WRN), which manifests in premature aging like graying and thinning hair, bilateral cataract formation, atherosclerosis, and cancer predisposition. WRN is a G4 helicase that prevents the shortening of the telomeres, which form G-quadruplex structures. In WRN deficient cells, telomeres sequence is obscured due to impaired replication of G-rich strand (Damerla et al., 2012; Goto et al., 2013).
3. Fanconi anemia is a genetic human cancer susceptibility disorder. FANCJ is a structure-specific DNA helicase that dissociates DNA G-quadruplexes. Mutation in FANCJ gene causes ‘Fanconi Anemia,’ with the symptoms of bone marrow failure, predisposition to cancer, *etc.* (London et al., 2008; Wu et al., 2008).
4. Bloom syndrome is a rare autosomal recessive disease characterized by genomic instability, premature aging, primordial dwarfism, and predisposition to cancer, pulmonary diseases, *etc.* It is caused by BLM, the gene encodes a protein homologous to RecQ helicases and unwinds G4 DNA, and removes quadruplexes from switch regions to enable replication of switched B cells and robust immune response (Sun et al., 1998).

### Diseases associated with transcription helicases with G4-motifs

The mapping of the human genome revealed that the presence of G4-motifs near the promoter site helps to regulate the transcription by binding with the two most important transcription-associated helicases XPB and XPD. It has been determined that both the helicases are linked with 1.8% of the G4-motifs found in the human genome. This association of XPB and XPD with G4-motifs characterize specific signaling and regulatory pathways. These helicases (XPB and XPD) have been found important for nucleotide excision repair, and their specific mutant allele are associated with three human genetic disorders–xeroderma pigmentosum, trichothiodystrophy, and Cockayne syndrome. Xeroderma pigmentosum is characterized by damage from UV radiation, skin cancer, and melanoma. Trichothiodystrophy shows the symptoms of distinctive developmental defects, brittle hair, and physical and but no cancer predisposition, while developmental defects and progressive neurological degeneration are the symptoms identified for Cockayne syndrome (Gray et al., 2014).

### Neurodegenerative disorders due to G4 expansion repeats

The fragile X syndrome is an intellectual disability disorder caused by expansion of more than 200 repeats of CGG at 5′ untranslated region of FMR1 gene. If the repeat number is between 55 and 200, it is referred to as a permutation allele that does not cause FXS phenotype but is prone to increase in repeat length during meiosis. Though there are runs of 2 G’s, they have been found to form G-quadruplex. The symptoms include behavioral problems and anticipation. The sternness of the disease and the age of onset of the disease decrease from one generation to the next generation due to increase in the number of repeats. (Santoro et al., 2012; Maizels 2015).

Another example of G4 expansion is GGGGCC repeat in C9ORF72 gene at chromosome 9, whose sequence motif has been shown to form G-quadruplex (Haeusler et al., 2014), associated with two neurodegenerative diseases, namely, amyotrophic lateral sclerosis (ALS) and frontotemporal dementia (FTD) (DeJesus-Hernande et al., 2011; Renton et al., 2011; Rademakers 2012). The symptoms of ALS include stiff muscles, difficulty in speaking, breathing, swallowing, and respiratory failure, while FTD shows the symptoms of dementia, compulsive behavior but not loss of memory like Alzheimer’s disease (Maizal 2015). The number of GGGGCC repeats reported pathological is 30 but can be expanded further in some cases.

The two other neurodegenerative diseases, that is, primary progressive aphasia (Simón-Sánchez et al., 2012) and depressive pseudo depressive dementia (Bieniel et al., 2014) have also been found to be associated with GGGGCC repeat expansions but the reason for identical pathology with distinct symptoms is yet to explore.

### G-quadruplexes and their therapeutic effects

Thus an understanding of the diverse roles of G-quadruplexes in the biological system not only provides critical insights into fundamental molecular mechanisms that control cellular processes but also provides opportunities to identify novel therapeutic targets to treat injury and disease. Hence, an abundance of diverse G-quadruplex topologies and their biological implications have been well reported. The literature is rich in the evidence, showing the prevalence of G-quadruplex in the cellular system and its involvement in various diseases like neurological disease, cancer, and aging. This has proven these exotic structures a promising target for a therapeutic drug design (Teng et al., 2021). The structural heterogeneity of G4 DNA and their contribution in regulating biological processes replication, transcription of disease-related genes (c-myc, BCL-2, KRAS, and c-KIT), telomere maintenance etc. has been summarized (Teng et al., 2021). Similarly, Sanchez-Martin and others gave a nice detail of interaction of ligands with DNA and RNA G-quadruplexes, in cancer therapy (Sanchez-Martin et al., 2021).

The c-myc promoter is one of the most studied gene to understand the structure and biology of G-quadruplexes. The structural topology exhibited by c-myc G-quadruplex has been depicted as parallel stranded (Phan et al., 2004) c-myc, an oncogene, control the cell growth as well as apoptosis. Its overexpression leads to human malignancies like colon, breast, prostate, and cervical carcinomas. It has been found that nuclease hypersensitivity elements-III (NHEIII_1_) and the G-quadruplex structure are important for c-myc transcriptional silencing (Siddiqui-Jain et al., 2002; Thakur et al., 2009; Brooks and Hurley 2010). Thus G-quadruplex stabilizing ligand reduces c-myc expression and are called antitumorigenic (Grand et al., 2002; Ou et al., 2007).

Likewise, BCL-2 promoter, a mitochondrial membrane protein acts as inhibitor of cell apoptosis. When over expressed, it leads to different kind of human tumors like B cell and T cell lymphomas, breast, prostate, and cervical carcinomas (reviewed by Yang and Okamoto 2010). BCL-2 is a GC-rich promoter with multiple transcriptional start site located within a nuclease hypersensitive site. The G-rich strand found in this region has been shown to form intramolecular mixed parallel/antiparallel G-quadruplex with both reversal side loop and lateral loop (Dai et al., 2006). Similarly, the G-quadruplex formation in other human promoter gene like VEGF, KRAS, and C-KIT has been nicely reviewed by Yang and Okamoto (2010).

A large number of G4 ligands like porphyrin derivatives, acridine derivatives, pyridine derivatives, and natural alkaloids have been tailored to act as anticancer therapeutics by stabilizing G-quadruplexes and have been summarized elegantly by Teng et al. For instance, the potential G4 ligands reported for c-myc includes TMPy4, BRACO-19, and pyridostatin, which suppresses the growth and proliferation of breast cancer cells, retinoblastoma cells, and melanoma cells (Mikami-Terao et al., 2009; Rapozzi et al., 2014; Asamitsu et al., 2019; Konieczna et al., 2019).

Furthermore, multiple telomeric G4 targeting ligands have been reported, which inhibit telomerase activity and thus tumor growth. Telomestatin, a telomeric G4 binding ligand is a natural compound obtained from *Streptomycees anulatus*, which inhibit telomerase activity and shorten telomere length and show antiproliferative effects in acute leukemia and myeloma (Kim et al., 2003; Shammas et al., 2004).

Berberine has also been reported as telomere binding ligands. It possess anti-inflammatory activity for various chronic disease and anticancer properties against human cancer cells (Yu-Ru et al., 2020). Another application of berberine has been reported in a recent report where it has been demonstrated to target the promoter site also in DUX4 gene inhibiting muscle fibrosis. Furthermore, berberine derivatives have also been developed to improve the efficacy. Ber8, a 9-substituted berberine derivative shows string interaction with telomeric G-quadruplex and accelerate DNA damage in multiple cancer cells (Ciszewski et al., 2020).

Thus multiple studies have flooded the literature with diverse families of G4-interacting compounds, with improved affinity and specificity. Numerous other potential of G-quadruplex-interacting drugs including quarfloxin (Ki et al. JMC 2003), 307A (Lemarteleur et al., 2004), RHPS4 (Leonetti et al., 2004), perylene such as PIPER (Han et al., 1999), quindoline derivative (Zhou et al., 2005), berberrubine (Saha et al., 2019), and quercetin (Vinnarasi et al., 2019) have been documented.

Exploring various G-quadruplex stabilizing drugs have been proven significant for the development of anticancer drugs. For example, ligands like berberrubine (Saha et al., 2019), berberine (Suganthi et al., 2018), quercetin (Vinnarasi et al., 2019), and telomestatin (Kim et al., 2003) have been documented as DNA G-quadruplex stabilizing drugs possessing anticancer properties.

Moreover, the gene therapy of various diseases has also been studied by targeting G-quadruplexes by compounds like ranitidine chloride and benzophenanthridine derivative. The binding was studied with G-rich DNA sequences of HIV-1 promoter (Mitrasinovic et al., 2018). The *in vitro* interaction study of folic acid conjugated meso-silica nanoparticle with G-quadruplexes has provided a new paradigm for understanding anticancer activity (PoshtehShirani et al., 2018). Furthermore, another novel enantioselective approach of ligand binding with DNA G-quadruplex has taken the DNA-binding studies to an established approach (Park et al., 2018).

## Aptamers

Aptamers are single-stranded (ss) DNA or RNA molecules consist of 20–80 nucleotides that fold into a three-dimensional conformation and bind to specific targets. The word aptamer is derived from the Latin word aptus, meaning to fit, and the Greek word meros, meaning part. Aptamers are also termed as chemical antibodies, as their process of molecular recognition is identical of antibody–antigen interaction and can be synthesized by an *in vitro* evolution method, known as “Systematic Evolution of Ligands by Exponential Enrichment” (SELEX) (Ku et al., 2015; Yüce et al., 2018). Aptamers can specifically recognize and interact with a wide variety of targets such as small molecules, peptides, proteins (receptors), antigens, biomarkers, viruses, whole cells, and tissues *via* hydrogen bonding, electrostatic interactions, stacking interactions, and van der Waals forces (Hayashi et al., 2014; Kumar Kulabhusan et al., 2020). Their ability to bind specifically to the targets ranging from ions to the whole cells, as well as other special attributes such as ease in synthesis, low immunogenicity, excellent tissue permeability, good stability, and modifiability make them a potential candidate for diagnostic and therapeutic applications in medical, clinical, and immune hematological fields (Yang et al., 2018; Guan and Zhang 2020).

A comprehensively described state-of-the-art knowledge on the DNA-based aptamers targeting thrombin, their optimized analogs, and their biological performances in therapeutic applications is reviewed (Riccardi et al., 2021). Several known aptamers consist of G-rich sequences capable with significant biological activity are able to fold into peculiar G-quadruplex (G4) structures. Accordingly, a pentadecanucleotide, the best characterized thrombin-binding aptamer (TBA) so far, prone to form G-quadruplex, which is able to specifically recognize the protein exosite I, inhibiting the conversion of soluble fibrinogen into insoluble fibrin strands. Studies revealed that in thrombin-bound or unbound forms, TBA is able to form an antiparallel, intramolecular G4 structure, consisting of two G-tetrads connected through three edge-loops, that is, a central TGT and two lateral TT loops. The plethora of studies carried out on TBA in almost 30 years from its discovery revealed that loop residues T4, T9, T13, and G8 are critical to preserve the G4 structure, whereas T3, T7, and T12 are more flexible moieties and are particularly involved in the thrombin inhibition process (Adachi, and Nakamura 2019; Riccardi et al., 2021).

The process of making aptamer-integrated DNA nanostructure is an important challenging issue as its integrity is affected by many factors like pH, temperature, concentration of metal ion, and nuclease degradation. Several methods have been formulated to improve the stability, affinity, and specificity of these structures. For instance, ligation of 5′- phosphate group and 3′-OH group of two aptamers construct a circular bivalent aptamer, with enhanced enzymatic resistance to Exo 1 (Kuai et al., 2017).

Several aptamer-based DNA nanostructures have been devised to sense cells. Zuo and others developed tetrahedron-based apta-sensor to detect cancer cells in HeLa and MCF7 cells. With the help of multibranched hybridization chain reaction (HCR), gold nanoparticles *etc.,* high sensitivity, efficient signals could be achieved to capture and detect down 24 cancer cells (Zhou et al., 2014). Thus, the integration of aptamers with DNA nanostructures open a window to examine the specificity of various biomolecule present on/within the cell surface (Huang et al., 2021). Furthermore, some aptamers bind to specific receptors on the cell membrane as a probe to visualize it. For example, Wang et al. designed an aptamer probe capable of targeting membrane proteins of living cancer cells with clear imaging (Shi et al., 2011).

Aptamers binds to the misfolded (target) proteins and impede their accumulation in the central nervous system; hence, they have also been reported as treatment option for numerous neurodegenerative diseases including Alzheimer’s disease, Parkinson’s disease, Huntington’s disease, and multiple sclerosis (Ozturk et al., 2021).

With the aim of lowering side effects and high therapeutic effects, the targeted drug delivery system (DDS) has become one of the most important paradigms for the treatment of diseases. Hence, aptamer-based DNA nanostructure and aptamer drug conjugates have been emerged out promising candidate for biological and biomedical applications (Huang et al., 2021).

### i-motif structures

i-motif is another exotic example of diverse non-canonical DNA structures. Various aspects of this structurally and biologically relevant non-canonical DNA structural entity have elegantly been reviewed (Abou Assi et al., 2018). The complementary strand of guanine-rich sequences tends to form an interesting and novel four-stranded structure, termed the i-motif DNA (intercalated-motif DNA), consisting of two parallel cytosine-rich strands forming a homoduplex that is intercalated in an antiparallel orientation. Protonation at N3 of cytosine present in the C-rich sequence is required for the formation of an i-motif structure; however, stable i-motif structures can be formed even at neutral pH. Intriguingly, a novel motif structure, called AC-motif has been reported where the conventional C^+^:C base pairs intercalate with protonated adenine cytosine (A^+^:C) in the presence of Mg^2+^ at physiological pH (Hur et al., 2021). The pH-dependent formation of i-motif questioned the existence of this structure *in vivo* at physiological pH; however, the prevalence of the C-rich regions, especially within the human telomere and promoter region of the oncogenes, near or within the regulatory region of eukaryotic genomes, recommended its biological importance (Manzini et al., 1994; Huppert and Balasubramanian 2007; Brooks et al., 2010). Though these structures are thought to have regulatory functions, their *in vivo* existence has so far remained elusive. Recently, i-motifs have been detected in the nuclei of human cells using antibody fragments that recognize their structure specifically (Zeraati 2018). They also discovered that these structures mostly form at a particular point in the cell’s cycle–the late G1 phase, and hence are cell and pH-dependent. Using NMR, the stability of i-motifs inside living mammalian cells was demonstrated by Dzatko et al. (2018) confirming the existence of these exotic structures in the cellular environment. Biological processes may lead to localized changes in pH that can stabilize pH-dependent DNA structures, such as i-motif and Py-motif triplexes.

**FIGURE 13:**
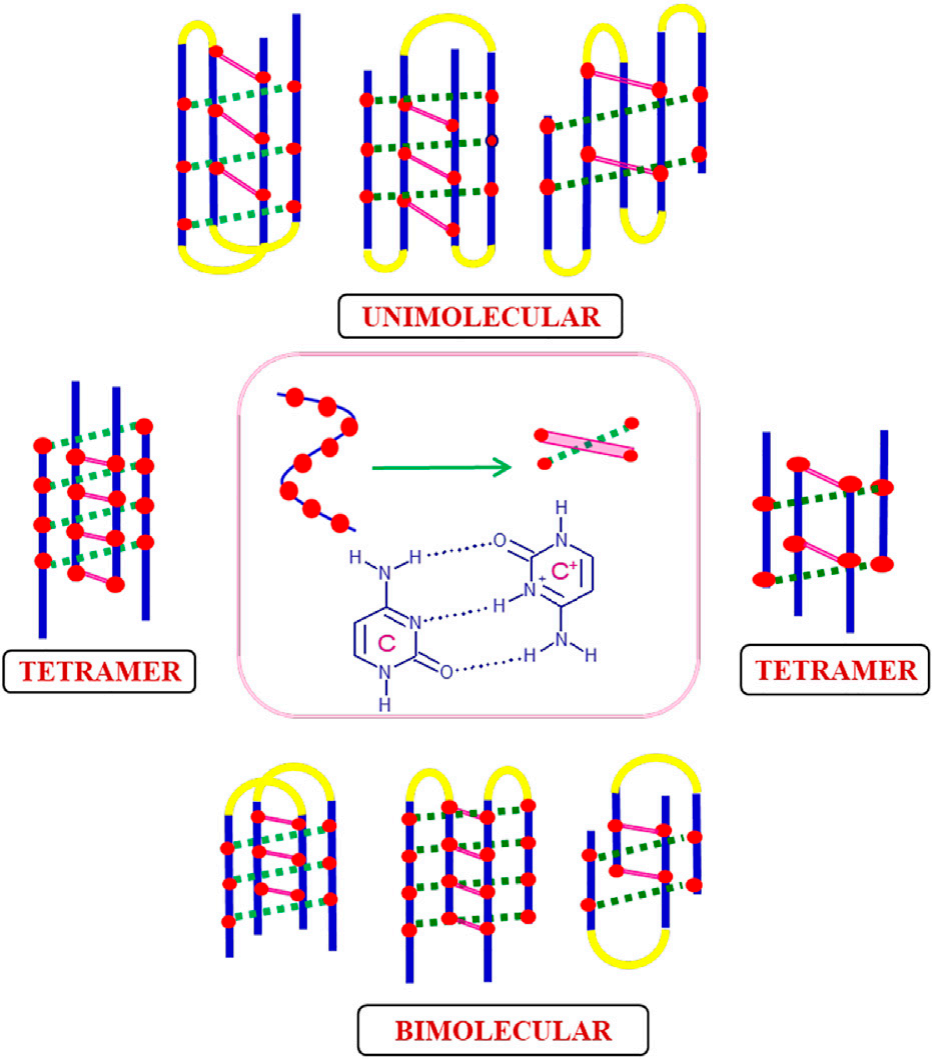
Different topologies of i-motif DNA structures.

Depending on the number of cytosine bases, solution conditions, and the loop length, i-motif structures with different molecularity like uni-, bi-, and tetramolecular have been reported as shown in Figure 13. The stability of these structures is also governed by modifying the sugar backbone of DNA; for instance, 2′-fluoro arabinonucleic acid modifications increase i-motif stability whereas substitution of cytidines with threoninol destabilizes i-motifs (Pérez-Rentero et al., 2015; Abou Assi et al., 2017).

The first intermolecular i-motif, for the sequenced (TCCCCC) was characterized by Gehring et al. (1993) under acidic conditions. Han et al. (1998) have shown that d (5mCCT_3_CCT_3_ACCT_3_CC) forms an i-motif structure that is stable even at neutral pH. Recently, the co-existence of i-motif and B-DNA, resulting in the formation of i-motif/duplex interfaces (or junctions) has been shown by NMR methods at neutral pH (Serrano-Chacón et al., 2021). Researchers have shown that i-motif is found to be structurally dynamic adopting different structures/conformations, ranging from a bimolecular complex to tetramolecular forms over a wide pH range. The structural status of the truncated double repeat of human telomeric sequenced (CCCTAACCC)), and its extended version, d (CCCTAACCCTAA) has been investigated by Kaushik et al. The truncated version was shown to adopt a bimolecular i-motif structure, whereas its complete double repeat (12-mer) sequence exists in two (bimolecular and tetramolecular) forms as revealed by pH-dependent UV-melting, gel-electrophoretic, and circular dichroism studies (Kaushik et al., 2010). A study on similar lines using laser tweezers techniques, the C-rich human ILPR sequence demonstrated that partially folded and i-motif forms can co-exist and can inhibit transcription catalyzed by RNA polymerase (Dhakal et al., 2010).

Several studies relating to i-motif provided numerous breakthroughs, which posit this structure as a target for therapeutic development, their ability to regulate nuclear processes like they can block DNA replication (Catasti et al., 1997; Brown and Kemdrick 2021). The credibility of i-motif as a biologically relevant structure for regulating gene expression is approved with the proximity of i-motif elements to the transcription start site. Depending on the specific promoter region, i-motif sequence, and the involved transcription factors show their capability to act as activators or repressors of transcription. There are reports of the i-motif structure formation, in the bone development proteins, sequence-specific DNA-binding domain transcription factors, and different gene encoding oncogene in the human genome (Kang et al., 2014; Kendrick et al., 2014; Delbridge 2016; Wright et al., 2017).

Research reports have highlighted various small molecules elucidating the function of i-motif as a therapeutic target and also facilitating drug discovery. For example, the porphyrin, TMPyP4, the first i-motif interactive molecule increases the stability of c-myc i-motif and prevents binding of hnRNPK (Bialis et al., 2007). However, it decreases the stability of BmPOUM2 i-motif and results in the downregulation of transcription (Niu et al., 2018). Similarly, in a recent report, the binding effect of flavonoids with BCL-2 i-motif was investigated where luteolin and quercetin was found to interact with BCL-2 i-motif with great affinity, giving the insight to understand the inhibitory condition of human cancer at the molecular level (Yang et al., 2020). Poly-C-binding proteins (PCBPs) consisting of hnRNPK (heterogeneous nuclear ribonucleoprotein K), CP1-4, and CP-KL proteins have been shown to interact with C-rich sequences and contribute a fundamental role in regulating gene expression (Yoga et al., 2012). Furthermore, carboxylated single-walled carbon nanotubes (SWNTs) were shown to selectively stabilize the telomeric i-motif and inhibit telomerase. The stability of i-motif by SWNTs results in uncapping of telomeres, DNA damage, and apoptosis or senescence. The mechanism of stabilization of i-motif by SWNTs is associated with the proton exchange between the carboxyl group on the SWNT and i-motif cytosine, which results in hemiprotonation of the sequence and thus the folding of a stable structure (Chen et al., 2012; Wolski et al., 2019). Thus, the literature is rich in reports about different ligands that stabilize or destabilize i-motifs, depending on the nature of the interaction (Sheng et al., 2017; Pagano et al., 2018).

The ongoing research on the formation, stability, and function of i-motif has recently highlighted the role of this non-canonical structure in Parkinson’s disease. On examining the α-syn protein that forms oligomers and amyloid fibrils linked to cell death of neurons. It has been determined that α-syn protein binds to both telomeric G-quadruplexes and i-motif but neuronal protein promotes the folding and stability of the only i-motif structure, not the G-quadruplex. Unfortunately, the mechanism driving Parkinson’s disease is not completely defined yet; however, an attempt to solve the puzzle of the interplay between the α-syn protein and i-motif in the regulatory regions of the important genes of this disease provides an insight into the pathological defects of other neurological diseases like bipolar disorder and schizophrenia (Knop et al., 2020).

The literature is scanty in reports on the 3D structure of any i-motif/ligand complex. Attempts for such studies will pave the way for the development of drugs based on i-motif recognition. Though the present knowledge strongly suggests the *in vivo* existence of i-motifs, it is believed that these structural states are transient in the cell. Therefore, more *in vivo* studies are needed to confirm i-motif formation in various cell cycle stages or at different transcriptionally active sites within the chromosomes.

## Conclusion and future outlook

The biological chemistry of DNA and RNA is facing a new aspect today. In this review, we strived to understand and summarize the current knowledge on various non-canonical structures formed by different DNA repeats and their connection with various human diseases (summarized in Table 3). Biological processes such as replication, transcription, reverse transcription, translational regulation, and genome stability are governed by non-canonical DNA structures formed within these disease-related genes. For almost last seven decades, biochemists have characterized and elucidated the various conformations of non-B-DNA structures like hairpin, cruciform, triplex, tetraplex, Z-DNA, and i-motif. These structures play a crucial role in almost all biologically imperative processes. It is now well understood that non-B-DNA-forming sequences induce the genetic instability and cause many rare, dominant, hereditary, and neurological disorders such as xeroderma pigmentosum, trichothiodystrophy, and Cockayne syndrome, Alzheimer’s disease, Parkinson’s disease, Huntington’s disease, Friedreich ataxia (FRDA), fragile X syndrome (FRAX), myotonic dystrophy, and certain types of spinocerebellar ataxia. Some of these structures also act as a signpost of an oncogene, and a regulator for the expression of oncogenes at the transcription level. The stabilization of these structures is important to inhibit/downregulate the expression of a gene associated with human diseases like cancer. This emerging area has implications for understanding the challenges posed by alternative DNA structures and the mutagenic mechanisms through which they alter genome stability. A recent interesting study on G-quadruplex and i-motif structures reveals that their formation in the human cells is interdependent. These non-canonical structures can be discriminately stabilized or destabilized, acting as gene regulatory controllers. The finding may have important implications as the interdependency of structures points toward the interplay as an important gene regulatory switch (King et al., 2020).

**TABLE 3:** Role of non-canonical structures in progression of cancer and neurological diseases.

Gene name	Expected structure	Location in genome	Biological role in disease	Reference
ADAM-12	Z-DNA	5′-UTR	Negative regulatory element (NRE) at the 5′-UTR of ADAM-12 act as transcription repressor and regulate ADAM-12 expression	Mochizuki et al. (2007)
SCL	Z-DNA	Alternating Pu-Py on chromosome 3	Translocation causes dysregulation of SCL gene function and cause human tumor	Aplan et al. (1992)
BCL-2	Z-DNA	Alternating Pu-Py region at 5′- hot spot region	Causes chromosome translocation in 5′-flanking region in the BCL-2 gene and dysregulation of BCL-2 gene function	Adachi and Tsujimoto (1990)
c-myc	Z-DNA	Promoter	Causes cancer	Wölfl et al. (1995)
FMR1	G-quadruplex	CGG at 5′-UTR	Causes fragile X syndrome due to expansion of CGG repeats	Maizels (2015)
C9ORF72	G-quadruplex	GGGGCC repeat in chromosome 9	Causes different neurodegenerative diseases, amyotrophic lateral sclerosis (ALS), and frontotemporal dementia (FTD)	Haeusler et al. 2014, DeJesus-Hernande et al. 2011
c-myc	G-quadruplex	Promoter	Overexpression leads to human malignancies like colon, breast, prostate, and cervical carcinomas	Brooks and Hurley 2010, Siddiqui-Jain et al. 2002
BCL-2	G-quadruplex	Mitochondrial membrane protein	Over expression leads to different kind of human tumors like B cell and T cell lymphomas, breast, prostate, and cervical carcinomas	Yang and Okamoto (2010)
VEGF	G-quadruplex	Promoter	Cancer	Teng et al. (2021)
KRAS	G-quadruplex	Promoter	Cancer	Yang and Okamoto (2010)
c-KIT	G-quadruplex	Promoter	Cancer	Yang and Okamoto (2010)
c-myc	Triplex	Promoter	The intramolecular triplex (H-DNA) formed interferes with transcription	Belotserkovskii et al. (2007)
Ha-ras	Triplex	Promoter	Triplex formation at Ha-ras gene specifically inhibits Sp1 binding and transcription	Mayfield et al. (1994)
HER-2/neu	Triplex	Promoter	Formation if intermolecular purine motif prevents protein binding resulting in transcription inhibition	Ebbinghaus et al. (1993)
Cyclin D1	Triplex	Promoter	Transcription inhibition is attained *via* antiparallel triplex formation using phosphorothioate TFOs	Kim and Miller (1998)
FXN	Triplex	Intron	Segment of the sequence containing GAA triplet repeat linked with Friedreich’s ataxia is shown to form a purine motif triplex	Rajeswari M.R. 2012

There is still much to know about non-canonical DNA/RNA structures. The dynamic and transient *in vivo* nature of these diverse non-canonical/non-B-DNA structures in the cellular molecularly crowded environment has always been a challenge for targeting or recognizing these structures. Even though a minor population of conformations is not often represented in structural studies, they may contribute to stability, folding, and functions. Conformations like Z-DNA would be of special interest as they may alter the readout of sequence information from the genome. The structure and function of Z-DNA will continue to create many surprises. Long Z-DNA containing genes, augmented for disease-causing mutations are yet to be fully explored. Structure-specific proteins/peptides and drugs/ligands have to be discovered to recognize specific non-canonical DNA/RNA structures to elucidate their roles in different biological processes. Since these structures have appeared as potential therapeutic targets for various genetic diseases, searching for the proteins or ligands, which could discriminate the DNA/RNA sequence and their actual structures would help the therapeutic strategy and drug discovery.

The structure and function of non-canonical DNA will continue to spawn many surprises. An insightful future goal is to screen such small molecules, which act as promising drug candidates for the treatment of the ailment by downregulating the expression of the disease-causing gene. Hope the aforementioned discussions foster a new interest in the paradigm of DNA non-canonical structural polymorphism, stabilization, and their implication in understanding the role in different biological processes.
